# Li_5_NCl_2_: A Fully-Reduced, Highly-Disordered Nitride-Halide Electrolyte for Solid-State Batteries with Lithium-Metal Anodes

**DOI:** 10.1021/acsaem.2c03551

**Published:** 2023-01-27

**Authors:** Victor Landgraf, Theodosios Famprikis, Joris de Leeuw, Lars Johannes Bannenberg, Swapna Ganapathy, Marnix Wagemaker

**Affiliations:** Faculty of Applied Sciences, Delft University of Technology, 2628 Delft, The Netherlands

**Keywords:** fully-reduced electrolyte, lithium nitride halide, Li_5_NCl_2_, lithium nitride, stability against Li metal

## Abstract

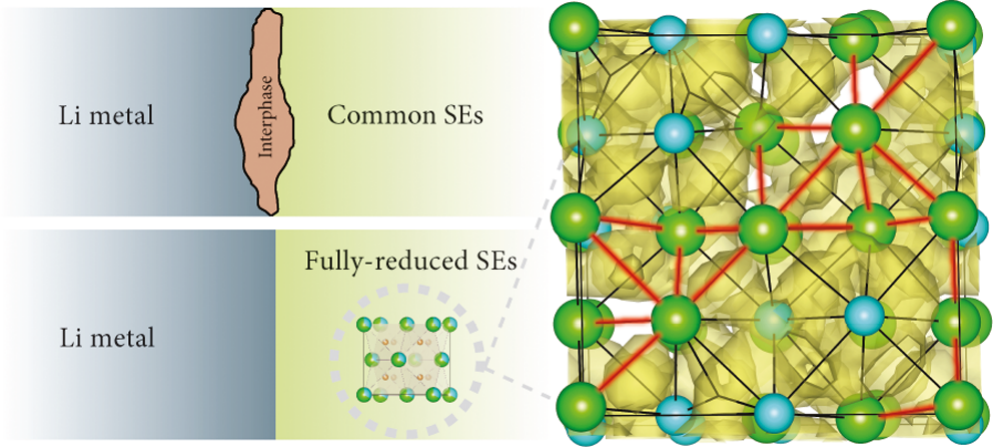

Most highly Li-conducting solid electrolytes (σ_RT_ > 10^–3^ S cm^–1^) are unstable against lithium-metal and suffer from detrimental solid-electrolyte decomposition at the lithium-metal/solid-electrolyte interface. Solid electrolytes that are stable against lithium metal thus offer a direct route to stabilize lithium-metal/solid-electrolyte interfaces, which is crucial for realizing all-solid-state batteries that outperform conventional lithium-ion batteries. In this study, we investigate Li_5_NCl_2_ (LNCl), a fully-reduced solid electrolyte that is thermodynamically stable against lithium metal. Combining experiments and simulations, we investigate the lithium diffusion mechanism, different synthetic routes, and the electrochemical stability window of LNCl. Li nuclear magnetic resonance (NMR) experiments suggest fast Li motion in LNCl, which is however locally confined and not accessible in macroscopic LNCl pellets via electrochemical impedance spectroscopy (EIS). With ab-initio calculations, we develop an in-depth understanding of Li diffusion in LNCl, which features a disorder-induced variety of different lithium jumps. We identify diffusion-limiting jumps providing an explanation for the high local diffusivity from NMR and the lower macroscopic conductivity from EIS. The fundamental understanding of the diffusion mechanism we develop herein will guide future conductivity optimizations for LNCl and may be applied to other highly-disordered fully-reduced electrolytes. We further show experimentally that the previously reported anodic limit (>2 V vs Li^+^/Li) is an overestimate and find the true anodic limit at 0.6 V, which is in close agreement with our first-principles calculations. Because of LNCl’s stability against lithium-metal, we identify LNCl as a prospective artificial protection layer between highly-conducting solid electrolytes and strongly-reducing lithium-metal anodes and thus provide a computational investigation of the chemical compatibility of LNCl with common highly-conducting solid electrolytes (Li_6_PS_5_Cl, Li_3_YCl_6_, ...). Our results set a framework to better understand and improve highly-disordered fully-reduced electrolytes and highlight their potential in enabling lithium-metal solid-state batteries.

## Introduction

Conventional lithium-ion batteries are reaching their theoretical limits in terms of energy density and rely on flammable liquid electrolytes.^1,2^ A promising alternative for the next generation of energy storage devices is all-solid-state batteries (ASSBs), which may enable the next step up in terms of energy density and safety required for the ongoing energy transition and the electrification of transport.^2,3^

Numerous solid electrolytes (SEs) have been developed, reaching Li-ion conductivities of up to 10^–2^ S cm^–1^, which are comparable with liquid electrolytes.^4−6^ However, the interfacial stability of SEs with both lithium metal (LM) and common high-voltage cathodes remains a tremendous challenge and hampers their application in ASSBs.^4,7,8^ A LM anode may be indispensable for ASSBs to surpass conventional lithium-ion batteries in terms of energy density;^3^ thus, stable LM/SE interfaces are crucial for the full-scale commercialization of ASSBs.

The stability of the LM/SE interface can be separated into two interlinked properties: (electro)chemical and (mechanical) contact stability. Chemical stability may potentially be achieved in two ways. (i) In the simplest case, the SE is thermodynamically stable against the LM, and no interphase is formed. (ii) If the SE is thermodynamically unstable against the LM, chemical stability can be achieved if it decomposes into an electronically insulating and ion-conducting interphase, self-limiting further decomposition, and thus effectively serving as a passivation layer^4,9,10^ Such interphases, however, may lead to inhomogeneous Li plating, which favors the growth of lithium dendrites and cell short-circuiting. Additionally, volume changes during decomposition may incite contact losses.^7^ It was previously believed that SEs could inhibit the growth of Li dendrites due to their high stiffness (large elastic moduli) compared to LM.^11^ This assumption has now been refuted theoretically^12^ and experimentally.^13,14^ Recently, the crucial role of microstructural aspects in inhibiting Li dendrite growth has also been established.^2^ This convoluted interplay of mechanical and microstructural properties, dendritic growth, and (electro)chemical and contact stability renders the design of stable LM/SE interfaces a difficult task. Uncontrollable SE decomposition at the LM/SE interface poses an additional engineering challenge and motivates the investigation of SEs that are thermodynamically stable against LM, as they may facilitate the stability of the LM/SE interface.

In search of new SEs, researchers investigated the compositional space between Li_3_N and LiX (X = Cl, Br, and I).^15^ As Li_3_N and the LiX salts are thermodynamically stable against LM, all members of the quasi-binary Li_3_N-LiX phase cuts were equally expected to be stable against LM. The numerous new phases discovered are called lithium nitride-halides. Cubic Li_5_NCl_2_ (LNCl), which crystallizes in the antifluorite structure, emerged as the best lithium-ion conducting lithium nitride halide (σ_RT_ = 1 × 10^–3^ mS cm^–1^),^15^ demonstrating excellent (electro)chemical stability against LM, an anodic limit of >2 V (vs Li^+^/Li), and low electronic conductivity of <1 × 10^–10^ S cm^–1^.^15^ Marx and co-workers revisited numerous lithium nitride halides, better refined their structures, and often corrected the initially reported stoichiometries.^16−19^ LNCl was found to have a stoichiometry of Li_5_NCl_2_, not Li_9_N_2_Cl_3_ as originally proposed.^17^ Thereafter, LNCl was not revisited until Galvez-Aranda and Seminario investigated the solid-solid LM/LNCl interface with ab-initio molecular dynamics (AIMD) simulations and confirmed the stability of the interface.^20^ Sang and co-workers recently performed an ab-initio high-throughput investigation and identified new lithium nitride halide phases that may potentially be synthesizable, of which some are predicted to be highly Li-ion conducting (>10^–4^ S cm^–1^).^21^

**Figure 1:**
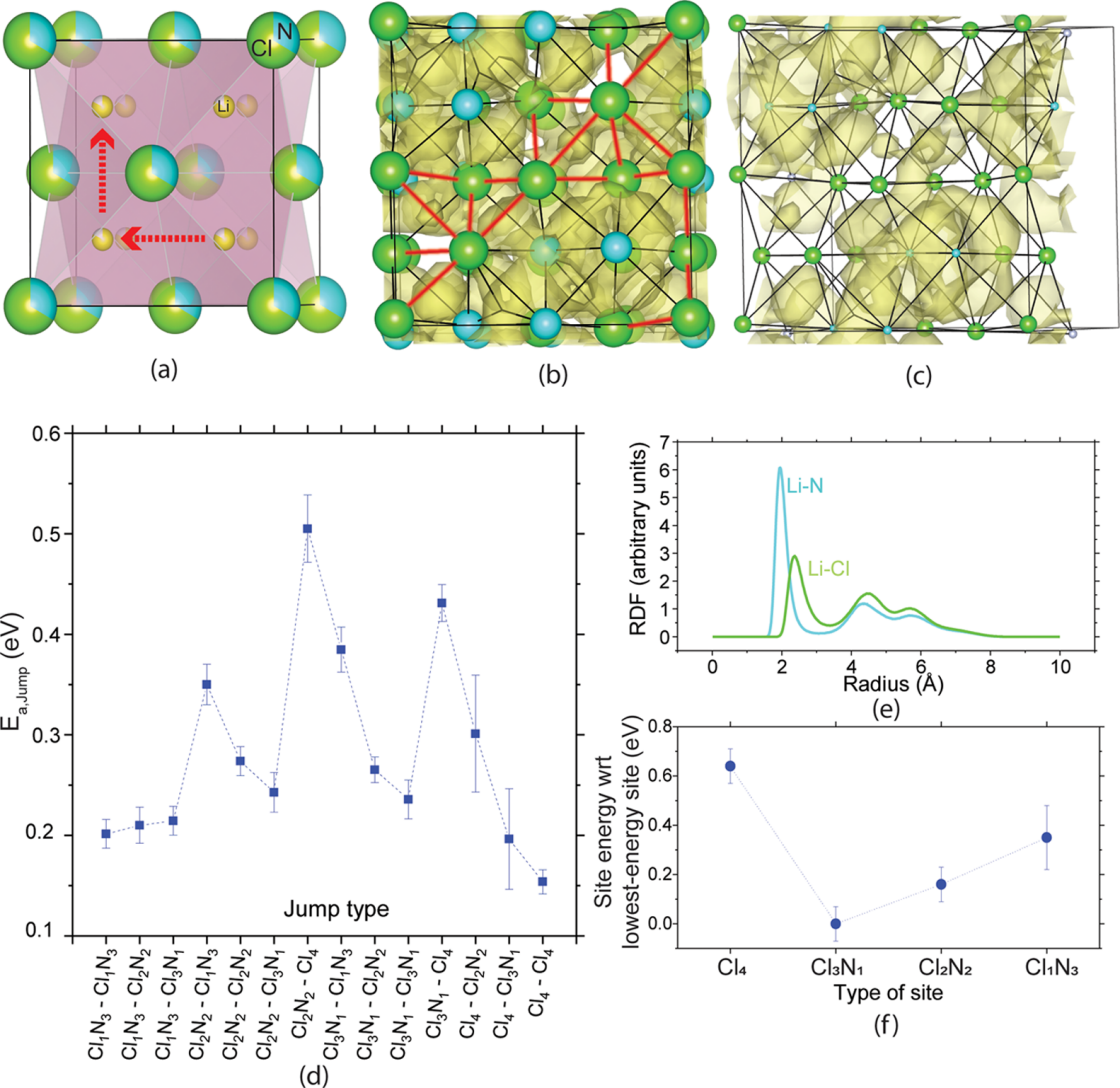
(a) Unit cell of LNCl. The green and turquoise spheres represent Cl and N, respectively. The red arrows schematically show the Li diffusion pathways in LNCl. (b) LNCl supercell with the Li density maps obtained from an AIMD simulation at 910 K. The tetrahedra surrounding Li sites are shown as black lines, and edges composed of two chlorides (Cl–Cl) are highlighted in red. (c) Same supercell as in (b). For better readability, only one layer is shown, the supercell is slightly turned, and the N/Cl ions are made smaller. (d) *E*_a,jump_ for different Li jumps. The average *E*_a,jump_ values, and their respective standard errors plotted here are obtained from seven AIMD simulations at 910, 860, 800, 720, 700, 680, and 650 K, respectively. (e) The RDFs obtained from an AIMD simulation at 910 K. (f) Relative energies of the different Li-sites, as obtained from the Li vacancy displacements to different sites.

LNCl crystallizes in the antifluorite structure with the *Fm*3̅*m* space group. N/Cl share occupation of the Wyckoff 4a (0,0,0) site with a 1:2 ratio (Figure 1a).^17^ The tetrahedral interstitials (Wyckoff 8c (0.25, 0.25, 0.25)) are partially occupied by lithium ions (82.5%). LNCl thus features a partially occupied lithium sublattice, which is a good predisposition for high lithium-ion conductivity.^22^ Reinvestigating materials with promising structural features recently led to the discovery of high ionic conductivities in materials that were thought to be poorly conducting. Lithium halide ceramics with trivalent metals (Li_3_MX_6_, M = Y, Er, Zr, In...; X = Cl, Br, and I), for example, had been known for decades.^23^ However, their high ionic conductivities were only discovered after Asano and co-workers in 2018 first demonstrated that ionic conductivities in the range of 0.03–1.7 mS cm^–1^ could be obtained for Li_3_YCl_6_ and Li_3_YBr_6_ via a mechanochemical synthesis route.^24,25^ In contrast, reinvestigating the electrochemical stability window of SEs, showed that previously reported stability windows were frequently too large; the stability window of SEs was systematically overestimated because of the use of semi-blocking electrodes that provide poor contact at the SE/electrode interface.^26,27^

In view of the above and because of its excellent stability against LM it is due time to reinvestigate LNCl. Combining experiment and calculation we investigate (i) the fundamental bulk ion-conduction mechanism in LNCl, (ii) the effects of mechanochemical treatments on LNCl, and (iii) its anodic limit. Based on our findings, we aim to map out potential applications of LNCl in ASSBs.

## Results and Discussion

### Computational Investigation of Li Diffusion in LNCl

LNCl is a material with partial occupancies, and constructing a 2 × 2 × 2 supercell that can be used in ab initio\simulations necessitates the probing of different possible atom arrangements. Inspired by refs (28) and (29), we obtained a model supercell of LNCl by a combination of electrostatic energy minimization and the screening >10,000 atom arrangements. We adopted the supercell with the lowest internal energy as our model LNCl supercell. Our model supercell is slightly distorted from cubic symmetry (Table S1), which is likely a consequence of its limited size. Similar distortions in the cubic symmetry of an LNCl supercell have been observed in a previous computational investigation (Table S1) of LNCl.^20^ Overall, good agreement is found between the unit cell parameters measured from Rietveld refinement of X-ray diffraction (XRD) data and our model supercell (Table S1). The side lengths of the supercell deviate by at most 2.5% and the supercell volume and density differ by less than 0.6% from experiments (Table S1). In LNCl, the Li sites are enclosed by tetrahedra composed of nitride and chloride ions. The tetrahedra are composed of either one nitride and three chlorides (from here on referred to as a Cl_3_N_1_ site), two nitrides and two chlorides (Cl_2_N_2_), three nitrides and one chloride (Cl_1_N_3_), four chlorides (Cl_4_), or four nitrides (N_4_). In mixed N/Cl tetrahedra, we found that Li was displaced from the center of the tetrahedron towards the nitride ions (Figure S1). Figure 1e shows the radial-distribution function (RDF) of the Li–N and Li–Cl distances throughout an ab-initio molecular dynamics (AIMD) simulation. The Li–N peak in Figure 1e appears at shorter radii than the Li–Cl peak, suggesting that a shorter Li–N distance is maintained not only in the fully relaxed state at 0 K (Figure S1) but also during diffusion between sites throughout the AIMD simulation. Figure 1b,c are lithium density maps of our AIMD simulations at 910 K. The density maps show that Li diffuses through the shared edge of two neighboring tetrahedra. The density maps also reveal that the Li density through shared edges composed of two chloride ions (Cl–Cl edges, red highlighting in Figure 1b) is lower than through Cl–N and N–N edges. This suggests that diffusion through N–N and Cl–N edges are more favorable than through Cl–Cl edges.

As done in previous studies,^30,31^ we dissected our AIMD simulations into individual jumps between lithium sites. This enabled us to estimate the average attempt frequency ν* in LNCl (1.08 × 10^13^ Hz) and the jump frequency between different sites ν_A→B_ as explained in detail elsewhere.^30^ The activation energy for a jumping event was estimated with the following expression
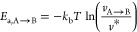
1where *k*_b_ is Boltzmann’s constant, *T* is the temperature in K, and *E*_a,A→B_ is the activation energy of a generalized jump event from site A to site B. We would like to highlight that a site-independent, isotropic attempt frequency of 1.08 × 10^13^ Hz is assumed. *E*_a,jump_ calculated with eq 1 can thus not be interpreted as the actual energy barrier for diffusion between two sites. *E*_a,jump_ should rather be interpreted as a metric for the propensity for jumps between two sites. The propensity for jumps between two sites is also represented by the jump frequency *v*_A→B_ but we favor the use of *E*_a,jump_ over *v*_A→B_ because *v*_A→B_ is temperature-dependent. The temperature dependence of *v*_A→B_ is moderated by the temperature term *T* in eq 1, making *E*_a,jump_ a temperature-independent metric for the propensity for jumps between two sites.

Figure 1d shows the activation energy *E*_a,jump_ for individual jump events in LNCl. The *E*_a,jump_ values in LNCl span a wide range from 0.2 to 0.5 eV, and it becomes apparent that jumps originating from Cl_2_N_2_ and Cl_3_N_1_ sites towards Cl_4_ sites show particularly high *E*_a,jump_ values (>0.4 eV). Figure 1f shows the energy differences between the Li sites. This energy difference between Li sites was obtained by displacing a Li-ion in site A to a vacant site B. We then interpreted the energy difference between the two supercells as the energy difference between sites A and B. It becomes apparent that Cl_4_ sites are >100 meV higher in energy than all other sites. The large *E*_a,jump_ values for Cl_2_N_2_ → Cl_4_ and Cl_3_N_1_ → Cl_4_ jumps thus likely originate from the large site-energy differences between Cl_2_N_2_/Cl_3_N_1_ and Cl_4_ sites.

In LNCl Li diffuses through the edge of edge-sharing tetrahedra and thus diffuses through the bottleneck edges that are composed of either two chlorides (Cl–Cl), one chloride and one nitride (Cl–N), or two nitrides (N–N). To deconvolute the effects of the jump type (Cl_2_N_2_ → Cl_3_N_1_, Cl_2_N_2_ → Cl_2_N_2_, ...) and of the bottleneck composition on *E*_a,jump_, we focused on jumps between the same type of sites through different bottleneck compositions (Table 1). Examining jumps between Cl_3_N_1_ and Cl_2_N_2_ sites, respectively, we found that the more nitrogen the bottleneck contains, the lower *E*_a,jump_ (Table 1). This observation suggests that the *E*_a,jump_ values are affected by an intrinsic characteristic of the bottleneck.

**Table 1:** *E*_a,jump_ Values for Jumps between Equal Sites Through Different Types of Bottlenecks and the Average of all Types of Jumps Through Different Types of Bottlenecks^a^

	*E*_a,jump_ through different bottlenecks (eV)		
type of jump	Cl–Cl	Cl–N	N–N
Cl_3_N_1_ → Cl_3_N_1_	0.41 ± 0.03	0.23 ± 0.01	
Cl_2_N_2_ → Cl_2_N_2_	0.43*	0.34 ± 0.02	0.21 ± 0.01
Cl_1_N_3_ → Cl_1_N_3_			0.18 ± 0.01
Cl_4_ → Cl_4_	0.13 ± 0.01		
average of all types of jumps	0.32 ± 0.02	0.27 ± 0.01	0.22 ± 0.01

a Note: The averages and their respective standard errors listed in this table are obtained from three AIMD simulations at 910, 860, and 800 K. Cl_2_N_2_ → Cl_2_N_2_ jumps through Cl–Cl bottlenecks were only observed at 910 K and only between one of the two Cl_2_N_2_ → Cl_2_N_2_ site pairs that are connected by a Cl–Cl bottleneck (Table S5), which is why we do not report a standard error for this value (marked with *).

Bottleneck size is often considered to influence the activation barrier.^22,32,33^ We define the bottleneck size in LNCl as such
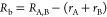
2where *R*_A,B_ is the distance between the peripheral atoms, and *r*_A_ and *r*_B_ are the ionic radii of the peripheral ions A and B. In the optimal case, *R*_b_ should be equal to the diameter of Li^+^, which is 1.18 Å (in tetrahedral coordination).^22,34^ If the bottleneck is too large or too small, energy may be required to adjust the bottleneck size, which increases the activation energy for a jump process.^22^ Using the Shannon radii^34^ of Cl^–^ and N^3–^, we calculated the *R*_b_ for Cl–Cl, Cl–N, and N–N bottlenecks to be 0.10, 0.50, and 0.72 Å. In each case, *R*_b_ is smaller than 1.18 Å, and energy is likely required to open the bottleneck for Li^+^ diffusion. Interestingly, the Cl–Cl bottleneck with (for a fixed jump type) the largest *E*_a,jump_ has an *R*_b_ that is furthest from the optimum, and the N–N bottleneck with (for a fixed jump type) the lowest *E*_a,jump_ has an *R*_b_ that is closest to the optimum. Bottleneck size could thus provide an explanation for the *E*_a,jump_ dependence on bottleneck composition. We conclude from Table 1 that for the same jump type, altering the bottleneck composition affects *E*_a,jump_. In contrast, for the same bottleneck composition, *E*_a,jump_ varies for different jump types. Thus, *E*_a,jump_ depends in a convoluted manner on both, the jump type and the bottleneck composition.

From AIMD simulations of LNCl at different temperatures, we obtained the tracer diffusivity (*D*_tr_) at different temperatures, which enables the estimation of activation energy (Figure S2), as done in previous studies.^35^ The ion conductivity σ can be obtained from the diffusivity using the Nernst–Einstein equation^35^
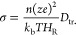
3where *n* and *z* are the number density of charge carriers and the charge of the charge carrier as a multiple of the elementary charge e, respectively. We assumed a Haven ratio *H*_R_ of 1. Our tracer-diffusivity analysis predicts an activation energy of 0.35 ± 0.03 eV and a conductivity of 0.3 mS cm^–1^ at 300 K for LNCl. Dissecting our AIMD simulations into individual jump events enabled the estimation of the jump-diffusivity *D*_J_, which can be obtained from the Einstein–Smoluchowski equation^30,36^
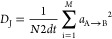
4where *N* is the total number of diffusing ions, *d* is the dimensionality of diffusion, *t* is the total simulation time, *M* is the total number of jumps observed, and *a*_A→B_ is the jump distance of a generalized jump-event. From *D*_J_, the conductivity can be estimated by inserting *D*_J_ into eq 3 instead of *D*_tr_. Our jump-diffusivity analysis predicts an activation energy of 0.27 ± 0.01 eV and a conductivity of 4.1 mS cm^–1^ at 300 K. Conductivities derived from *D*_J_ are typically larger than conductivities derived from *D*_tr_ because every jump is assumed to contribute to macroscopic diffusion. However, “back and forth” jumps between two sites and locally confined diffusion do not effectively contribute to macroscopic diffusion, and thus, tracer diffusivities better describe macroscopic bulk diffusion.

### Experimental Investigation of Li Conductivity in LNCl

**Figure 2:**
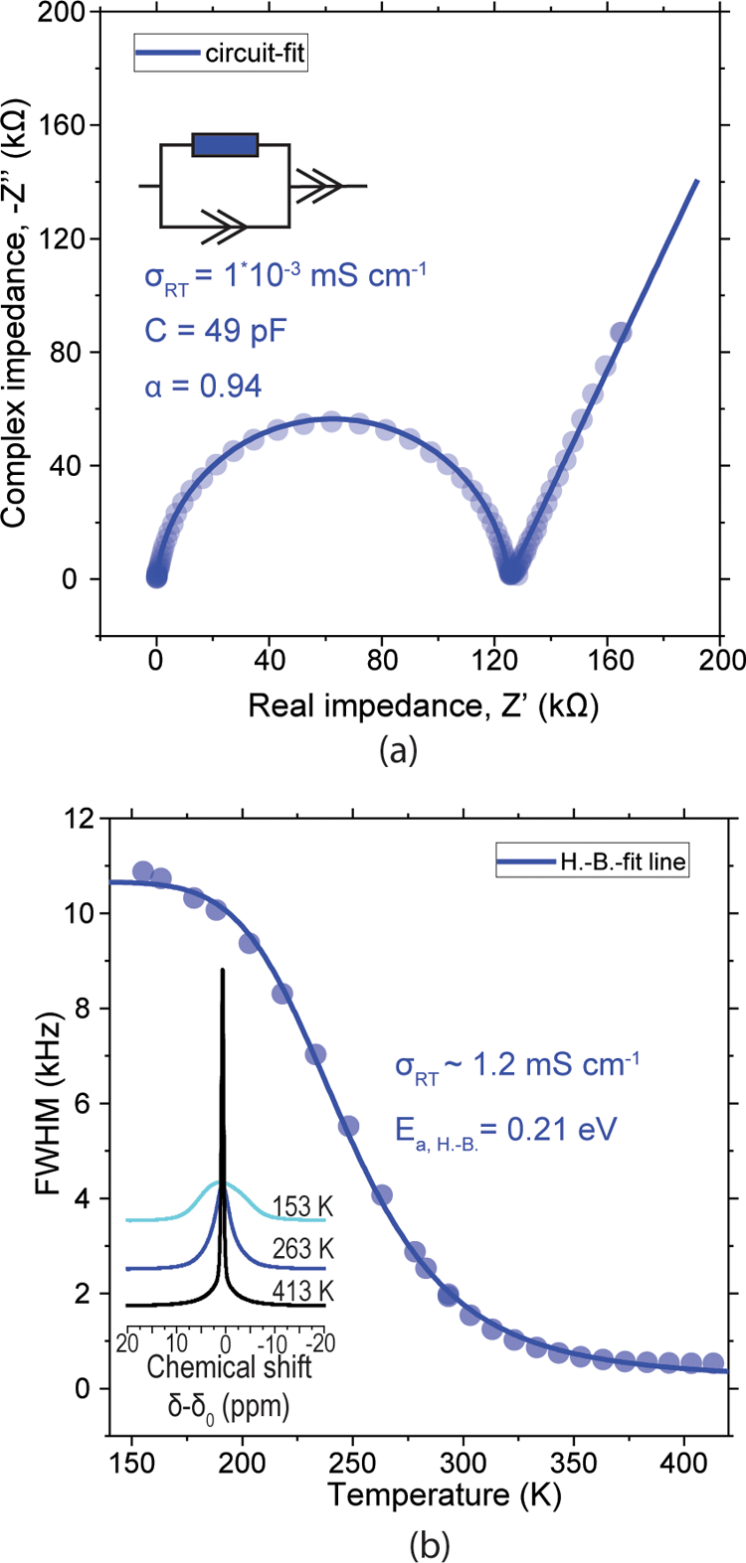
(a) Nyquist plot of LNCl-I at RT. Experiments were done in a SS|LNCl-I|SS cell (SS = stainless steel). The inset shows the equivalent circuit. (b) FWHM of the ^7^Li NMR signal of LNCl at different temperatures. The inset shows the NMR signal at three distinct temperatures. The solid line is the fit obtained from the Hendrickson–Bray equation.

We synthesized LNCl via a solid-state synthesis route by heating stoichiometric amounts of LiCl and Li_3_N at 600 °C for 3 h and subsequent air quenching, as shown in ref (37). LNCl synthesized in this way is from now on referred to as LNCl-I. The lattice constants previously reported for LNCl range from 5.386 to 5.416 Å.^17,38,39^ This variation in the lattice parameter may be a consequence of the different annealing protocols employed for LNCl synthesis in refs (17)(38), and (39). Also, the exact quenching procedure is not described in detail in refs (38) and (39). The lattice constant 5.396 Å that we determined from our XRD Rietveld refinements (Figure S3 and Table S1) for LNCl fits well into the range of lattice parameters determined in previous studies (5.386–5.416 Å).^17,38,39^ The purity of LNCl-I was verified by XRD, and no crystalline impurities were observed (Figure S3). Figure 2a shows the electrochemical impedance spectrum (EIS) Nyquist plot of LNCl-I at room temperature (RT). The impedance of LNCl-I could be fitted with a resistor (R) parallel to a constant phase element (CPE). The effective capacitance of the CPE calculated with Brug’s formula^40,41^ is 49 pF, which is a value typically associated with ion conduction in the bulk of solid ion conductors.^42^ A prominent secondary process in the impedance that may for instance arise from grain boundaries was not observed even at −30 °C (Figure S4). We thus interpret the impedance of LNCl-I at RT as bulk-dominated. We calculated the ionic conductivity of LNCl to be 1 × 10^–3^ mS cm^–1^ at RT, which is comparable with previous reports.^15^ The activation energy measured from temperature-dependent impedance spectroscopy was 0.471 ± 0.005 eV, which is close to the value of 0.49 eV previously reported for LNCl.^15^

Complementary to EIS, Li NMR provides insights on Li diffusion in ion conductors.^43^Figure 2b shows the ^7^Li NMR signal linewidth evolution with increasing temperature for LNCl-I, and the typical lineshape-narrowing profile was observed.^43−45^ The activation energy of the diffusion process that inflicts the line-narrowing can be obtained from the phenomenological equation derived by Hendrickson and Bray (H.-B.)^45^
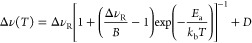
5where Δν(*T*) is the linewidth at temperature *T*, and Δν_R_ is the linewidth in the rigid-lattice regime. *B* is the linewidth that would be obtained at extreme narrowing in the absence of magnetic field inhomogeneity, and *D* is a correction factor accounting for broadening arising from the inhomogeneity of the static magnetic field. From this model, we obtain an activation energy of 0.21 ± 0.01 eV for lithium diffusion in LNCl. The value of B is an indicator of the “range” of motion. The larger *B*, the more “short-range” the motion because locally confined motion is not expected to entirely eliminate dipole–dipole interactions.^45^ For different ion conductors, values for *B* ranged from 10^–3^ to 10^–13^ kHz.^45^ The value of *B* obtained for LNCl (5 × 10^–4^ kHz) thus suggests that the line-narrowing is caused by locally confined motion.^45^ To what range exactly this motion may be confined, goes beyond the predictive scope of the Hendrickson–Bray equation.

An additional estimate of the activation energy can be obtained from the empirical expression of Waugh and Fedin (W.-F.)^46^
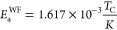
6where *T*_C_ is the onset temperature of motional narrowing in Kelvin. A drawback of the W.-F. approach is the difficulty to estimate the exact onset of motional narrowing. We observe the onset of narrowing at ∼180 K, and from the W.-F. expression, we obtain an activation energy of 0.29 eV. Generally, ^6,7^Li NMR measurements also capture Li motion that does not effectively contribute to macroscopic diffusion such as locally-confined diffusion and “back and forth” jumps. Activation energies obtained from *D*_J_ values thus better match activation energies obtained from ^6,7^Li NMR measurements than activation energies obtained with *D*_tr_ values. The activation energies obtained from the H.-B. and the W.-F. expressions and the activation energy obtained from the *D*_J_ values in our AIMD simulations are all in a range between 0.21 and 0.29 eV (Table 2). Generally, at temperatures below the onset of line narrowing, the jump frequency is lower than the rigid-lattice-regime NMR line width.^43^ At temperatures where the narrowing is observed, the jump frequency is higher than the rigid-lattice-regime NMR line width.^43^ When extrapolating the jump frequencies obtained from AIMD simulations to temperatures of the rigid-lattice regime (<150 K), we indeed obtain jump frequencies lower than the rigid-lattice-regime NMR line width of 10 kHz. Extrapolating the jump frequencies obtained from AIMD simulations to temperatures of narrowing (>180 K), we obtain jump frequencies >100 kHz, which are much larger than the rigid-lattice-regime NMR line width. We thus observe reasonable agreement between our AIMD simulations and NMR measurements.

**Table 2:** Ion Conductivity and *E*_a_ Obtained by Different Methods^a^

method	*E*_a_ (eV)	σ_RT_ (mS cm^–1^) [lower boundary; upper boundary]	length scale probed
AIMD_Tracer_	0.35 ± 0.03	0.3 [0.07; 1.2]	∼10 Å
AIMD_Jump_	0.27 ± 0.01	4.9 [4.4; 5.5]	∼10 Å
^7^Li NMR lineshape	0.25 ± 0.04	1.2 [0.18; 6.9]	<500 μm
EIS	0.471 ± 0.005	1.0 ×·10^–3^	∼500 μm

a Note: The lower and upper boundaries for σ_RT_ are obtained by using the respective extremes of the *E*_a_ values for extrapolation (e.g. 0.32 and 0.38 eV in the case of AIMD_Tracer_). For ^7^Li NMR line shape narrowing the lower and upper boundaries originate from different *E*_a_ values obtained from different models.

The activation energies obtained from NMR can be used to calculate the Li conductivity at 300 K in LNCl as follows^43^
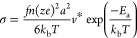
7where *a* is the average jump distance taken as 2.7 Å, which is the average distance between Li sites in LNCl, and *f* is the correlation factor; the correlation factor is a measure of how efficiently jumps contribute to macroscopic diffusion and can be calculated from the ratio *D*_tr_/*D*_J_.^30^ From the extrapolated values of *D*_tr_ and *D*_J_ (from AIMD) at 300 K, we obtained a correlation factor *f* = 0.06.

Using the range of activation energies obtained from the linewidth narrowing (0.21–0.29 eV), the correlation factor (*f* = 0.06), and assuming an attempt frequency ν* of 1 × 10^13^ Hz, a range for the ion conductivity at 300 K as probed by ^7^Li NMR was calculated (Table 2).

Table 2 summarizes the activation energies and RT conductivities obtained from different techniques. We observe reasonable agreement between our NMR measurements and the jump analysis of our AIMD simulations (Table 2). The activation energy obtained from the tracer analysis of our AIMD simulations is 0.08 eV larger than that of the jump analysis. This is most likely a consequence of the difference in how the mean square displacement is calculated (see Methodology), which may affect the respective temperature dependence of *D*_J_ and *D*_tr_. Diffusivities obtained from the jump-analysis (*D*_J_) tend to be overestimated because the jump-analysis captures locally confined motion that does not effectively contribute to macroscopic diffusion. Activation energies and conductivities obtained from the tracer-analysis (*D*_tr_) of AIMD simulations thus better compare to the bulk properties of materials measured by EIS. Interestingly, the activation energy obtained from EIS is 0.1 eV larger than the activation energy obtained from our AIMD tracer analysis. We would like to bring forward two conceivable explanations for this discrepancy. (i) The length scale probed by EIS is 5 orders of magnitude larger than the one probed in our AIMD simulations (Table 2). EIS may potentially capture a diffusion limitation occurring at the mesoscale of our LNCl pellets. Such a diffusion limitation may, for example, arise from potential amorphous impurities in our LNCl pellets (that would not be detected by XRD) and/or microstructural defects, as well as thin insulating surface layers on LNCl particles from a reaction with residual moisture in the glove box. Similarly, (ii) a second explanation for the discrepancy between our AIMD tracer analysis and our EIS experiments may be diffusion-limiting Cl_4_ sites and/or Cl–Cl edges in the bulk of our LNCl pellets: our AIMD analysis shows a poor propensity for jumps through Cl_4_-sites and Cl–Cl edges (*E*_a,jump_ > 0.4 eV). In our model, LNCl supercell (side length 10 Å) the Li diffusion pathways are not limited by Cl_4_ sites and/or Cl–Cl edges. However, with EIS we capture effects at the scale of our polycrystalline LNCl pellets (thickness ∼500 μm). Between different crystallites, the distribution of the Li sites likely varies, and the likelihood of diffusion-limiting Cl_4_ sites and/or Cl–Cl bottlenecks increases and may restrict the macroscopic conductivity, measured by EIS.

### Mechanochemical Treatment of LNCl

It has been repeatedly demonstrated that mechanochemical processing can dramatically improve the ion conductivity of inorganic ceramics.^25,44^

To investigate the potential benefits of mechanical milling, we processed LNCl-I for 4 h in a planetary ball mill after solid-state synthesis. Samples prepared in this way are from now on referred to as LNCl-I-BM. LNCl-I remains stable during the milling as no impurity peaks are detected in the XRD of LNCl-I-BM. The XRD of LNCl-I-BM shows dramatic peak-broadening compared to LNCl-I (Figure S5). Rietveld refinements show that the lattice parameter of LNCl-I is decreased from 5.39(6) to 5.37(6) Å after the mechanical milling process. Additionally, Williamson-Hall analysis (Figure S6) shows that after milling, the average crystallite size is dramatically reduced from the μm range to ∼60 nm. Milling also imposes a significant strain on the LNCl lattice so that we obtain a residual microstrain of ε = 1.04% for LNCl-I-BM.

**Figure 3:**
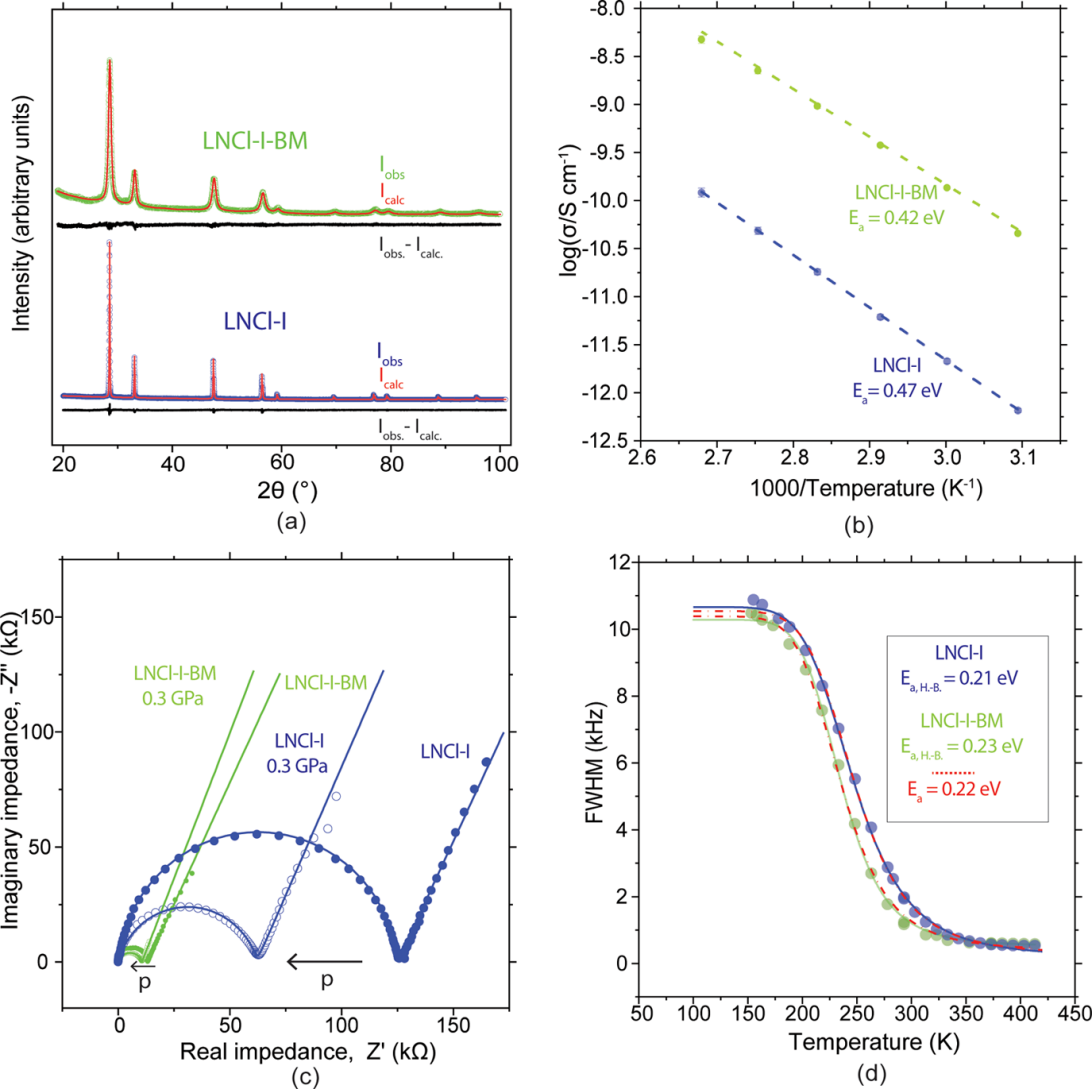
(a) XRDs of LNCl-I and LNCl-I-BM with Rietveld refinements and the difference profile. (b) Arrhenius fits of the conductivities obtained at different temperatures from EIS for LNCl-I and LNCl-I-BM. The individual points are the averages of three measurements, error bars are smaller than the point symbol and therefore not visible. (c) Nyquist plot of LNCl-I and LNCl-I-BM with and without pressure applied during measurement. (d) ^7^Li NMR signal line shape evolution with temperature for LNCl-I and LNCl-I-BM; the solid lines are the H.-B. fits, and the red dashed lines are obtained from H.-B. fits, with *E*_a_ as a fixed parameter set to 0.22 eV.

Interestingly, EIS shows that the ion conductivity of LNCl increases by an order of magnitude after milling reaching 0.01 mS cm^–1^. Additionally, fits of the conductivities at different temperatures to Arrhenius’ law (Figure 3b) show that the (macroscopic) *E*_a_ of LNCl-I is decreased from 0.471 ± 0.005 eV to 0.426 ± 0.005 eV after milling. As the crystallite size of LNCl-I-BM is substantially smaller, a larger volume fraction of grain boundary regions is expected in LNCl-I-BM, as compared to non-BM LNCl. However, the EIS of LNCl-BM-4 h could be excellently fitted with a single R-CPE with an effective capacitance of ∼40 pF. As for LNCl-I, we thus interpret the impedance in LNCl-I-BM to be bulk dominated. Longer milling of LNCl-I, for 8 and 12 h did not further increase the conductivity and no significant further decrease of the crystallite size was obtained (Figure S6).

Reduced particle size is a proposed explanation for the improved ion conductivity of mechanically processed materials.^47,48^ Under the same pelletization conditions, a smaller particle size facilitates compression and consolidation, which improves the area contact between particles and reduces the tortuosity for ion conduction.^47,48^ It was demonstrated that applying pressure during measurement on non-ball-milled (non-BM) Na_3_PS_4_ improved its observable conductivity by a factor of ∼10 so that it matched the conductivity of ball-milled (BM) Na_3_PS_4_.^48^ In contrast, applying pressure to the BM-Na_3_PS_4_ sample had a negligible effect on its conductivity. They concluded that the better conductivity of BM-Na_3_PS_4_ was a consequence of better compression/consolidation of the BM-Na_3_PS_4_ pellets used for EIS measurements.^48^ Inspired by this study, we investigated the conductivity of LNCl-I and LNCl-I-BM under pressure. We found that the conductivity of LNCl-I increased by a factor of ∼2 (Figure 3c). In contrast, the ion conductivity of LNCl-I-BM hardly improved when applying pressure. We thus explain our results as follows. In the case of LNCl-I, leeway for better consolidation was available, and we observed an increase in conductivity when applying pressure. For the LNCl-I-BM sample, the conductivity hardly improved because the smaller particle size enabled near-optimal consolidation during pelletization. However, two observations indicate that the improved conductivity of LNCl-I-BM is not solely a consequence of better consolidation. (i) Despite the pressure-induced conductivity improvement of LNCl-I by a factor of 2, even under pressure, the conductivity of LNCl-I-BM is still higher by a factor of 5. (ii) An improved conductivity solely originating from superior consolidation would not be expected to show a change in activation energy, but Arrhenius fits in Figure 3b clearly show that the activation energy for LNCl-I-BM is decreased by 40 meV as compared to LNCl-I.

Interestingly the Arrhenius prefactor of LNCl-I-BM is hardly larger than for LNCl-I. For LNCl-I-BM, we obtain log_10_(σ_0_/*S* cm^–1^) = 2.22 and for LNCl-I log_10_(σ_0_/*S* cm^–1^) = 2.05. The Arrhenius prefactor comprises factors such as the charge-carrier concentration, the attempt frequency, the jump distance, and the correlation factor. These factors are thus only slightly modified by milling, and the improved RT conductivity of LNCl-I-BM is predominantly a consequence of the decreased (macroscopic) activation energy. The origin of the reduced macroscopic activation energy of LNCl-I-BM is not entirely understood. Previous studies suggested that amorphous fractions in SEs introduced by high-energy milling may reduce the macroscopic activation energy and consequently improve ion conductivity.^44,46,49^ It was equally suggested that surface-related regions show faster ion diffusion due to increased structural disorder. An increased volume fraction of surface-related regions (concomitant with smaller particle size) may thus enhance bulk diffusion in nanocrystalline ceramics. Additionally, the mechanochemical synthesis may affect the local N/Cl ordering which may open-up lower-energy percolation paths in LNCl-I-BM.^44,46,50^

The activation energy of LNCl-I-BM obtained from a Hendrickson–Bray fit of the ^7^Li NMR lineshape narrowing profile is increased by 20 meV as compared to LNCl-I. This may suggest an increased activation barrier in LNCl-I-BM for the fast, locally confined Li diffusion probed by NMR. However, the accuracy of activation energies obtained from motional narrowing data relies on highly precise line-width measurements. Small errors in the line-width measurements may largely impact the fit result so that the uncertainty in activation energies obtained by this method typically amounts to a few tens of meV.^51^ This argument is visualized in Figure 3d, where a fit line obtained from an H.B.-fit with *E*_a_ as a fixed parameter set to 0.22 eV shows a good fit for the narrowing profile of LNCl-I and LNCl-I-BM.

To investigate the effects of further annealing on LNCl-I-BM, we reannealed LNCl-I-BM at 600 °C for 3 h and subsequent air-quenching restored the initial crystallinity of LNCl-I, and the conductivity was reduced to 1 × 10^–3^ mS cm^–1^. Annealing LNCl-I-BM also increased the activation energy to 0.486 ± 0.005 eV, which is close to the LNCl-I value of 0.471 ± 0.005 eV (Figure S7). We thus conclude that the beneficial effects of mechanochemical milling on LNCl-I are reversed when annealing the ball-milled samples. This result supports that the reduced particle size and the increased strain obtained after milling LNCl-I may potentially explain the increased Li conductivity of LNCl-I-BM.

Finally, we report that LNCl can be synthesized directly via a mechanochemical route without any annealing step. After milling stoichiometric amounts of LiCl and Li_3_N for 10 h at 600 rpm LNCl was obtained (Figure S7). Samples synthesized in this way are referred to as BM-LNCl. The RT conductivity of BM-LNCl was found to be 0.015 mS cm^–1^ and is the highest conductivity ever reported for LNCl. The activation energy of this sample is 0.416 ± 0.005 eV. Analogously to LNCl-I-BM, the conductivity of BM-LNCl is reduced to 5 × 10^–3^ mS cm^–1^ after annealing, and the activation energy is increased to 0.466 ± 0.005 eV, which are values comparable to well-consolidated LNCl-I (Figure S7).

### Electrochemical Stability Window of LNCl

**Figure 4:**
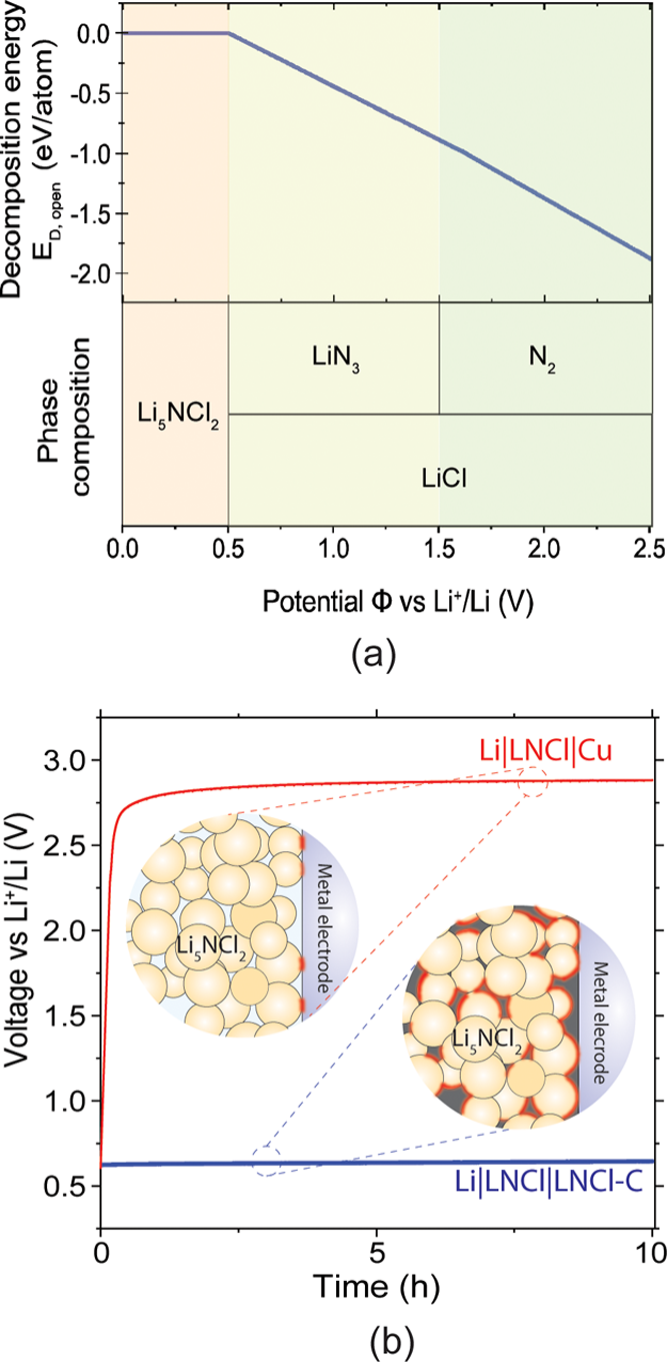
(a) Phase equilibria of the Li_x_NCl_2_ {*x* ∈ *R*|*x* ≥ 0} phase space at different potentials φ versus Li^+^/Li. Additionally, this figure shows the decomposition energy *E*_D,open_ as defined by Zhu, He, and Mo^52^ of LNCl at different φ. (b) Galvanostatic oxidation of a composite LNCl-C cathode and of LNCl in contact with an ion-blocking Cu disk. The two schematics represent the cathode compositions. In the case of LNCl-C composite cathodes, the contact area with the electronic network is much larger, as sketched by the red highlighting. The constant current for both curves in (b) was 0.5 μA (0.64 μA cm^–2^).

The anodic limit of LNCl was previously found to be >2 V versus (Li^+^/Li).^15^Figure 4a shows the results of our electrochemical stability calculations for LNCl, which were obtained by constructing grand potential diagrams at different chemical potentials of Li (μ_Li_) leveraging the materials project database.^52,53^ Our calculations predict that LNCl is thermodynamically stable against Li metal and has an anodic limit of 0.50 V versus (Li^+^/Li). Beyond 0.5 V, LNCl was predicted to decompose to lithium azide (LiN_3_) and LiCl (Figure 4a)

Overestimated anodic limits for SE were recently often reported.^27,54^ It was previously shown that employing an SE-carbon composite electrode in a Li|SE|SE-C cell instead of a simple ion-blocking metal (M) electrode in a Li|SE|M cell enabled a more accurate measurement of the anodic limit of SEs.^27,54^ The anodic limits obtained when using composite SE-C electrodes are typically significantly lower than the anodic limits obtained with ion-blocking metal electrodes. The smaller anodic limit measured is a consequence of the increased electrochemical surface area in a SE-C composite electrode; this increases the current signal at the onset of oxidation, making the onset of oxidation better observable.^27,54^Figure 4b shows the galvanostatic charge of a Li|LNCl|LNCl-C cell with an extremely low current of 0.5 μA (0.64 μA cm^–2^). The current onset at 0.62 V indicated an experimental anodic limit of 0.62 V for LNCl, which is in much closer agreement with the calculated value of 0.50 V than the previous experimental values of >2 V. We repeated the same experiment with a Li|LNCl|Cu cell, equally shown in Figure 4b; for this cell, the voltage is stabilized at 2.7 V, which may explain the overestimate in previous reports.

The predicted oxidation of LNCl to LiN_3_ calculated to occur at 0.50 V may potentially be kinetically inhibited even at very slow currents because LiN_3_ formation necessitates a complex anionic rearrangement to form the [N_3_]^−^ moieties present in azides. Such complex anionic rearrangements are additionally hampered by the likely limited RT diffusivity of Cl^–^/N^3–^ in LNCl. In the potential scenario of kinetically inhibited LiN_3_ formation, the calculated anodic decomposition voltage would be extended from 0.50 to 0.63 V versus (Li^+^/Li) (Figure S8), which is even closer to our experimental value of ∼0.6 V. In principle, the cathodic limit of SEs can be measured in an analogous way to the anodic limit. However, the use of composite SE-C electrodes makes it difficult to distinguish between the lithiation of carbon additives and the lithiation of the SE at potentials close to 0 V versus Li^+^/Li.^55^ Hartwig and co-workers reported the chemical stability of LNCl against LM.^56^ This stability was investigated by dipping LNCl into molten Li and the absence of any observed reaction.^56^ We repeated this experiment, keeping the LNCl powder submerged in molten Li at 210 °C for 2 h. No new peaks were observed after this in our XRD experiments (Figure S9). Additionally, symmetric Li|LNCl|Li cells display flat stripping/plating plateaus and no increase in the cell voltage (Figure S9) over time, indicating a stable LM/LNCl interface.

Based on our findings we now reflect on its applicability in ASSBs. A key property of LNCl is its thermodynamic stability against LM which is uncommon for SEs. However, its low anodic limit (∼0.6 V) inhibits its application in combination with common high-voltage cathodes and confines the applicability of LNCl to an artificial buffer layer between LM and alternative SEs that faces the cathode. In such a hybrid bilayer SE architecture, LNCl would be in close contact with a partner SE. The realization of such systems thus hinges on the chemical compatibility between LNCl and other common SEs.

**Figure 5:**
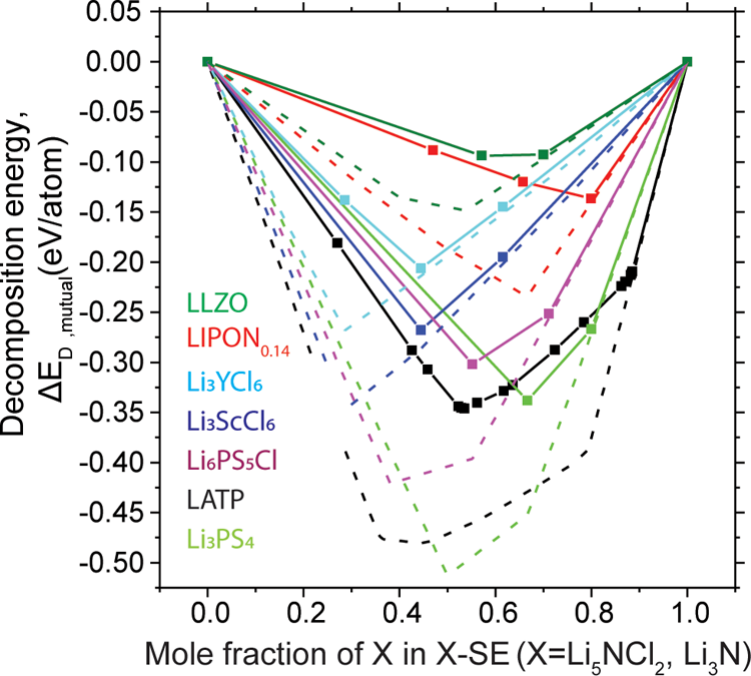
Pseudobinaries between LNCl/Li_3_N and common highly-conducting SEs. The solid lines are pseudobinaries between LNCl and different SEs and the dashed lines are for pseudobinaries between Li_3_N and different SEs.

We investigated the thermodynamic driving force of LNCl to chemically react with common SEs by constructing pseudobinaries in phase space (Figure 5), leveraging the materials project database, as explained in detail elsewhere.^52^Figure 5 shows the pseudo binaries between LNCl and different SEs. Δ*E*_D,mutual_ as defined by Zhu, He, and Mo^52^ is the reaction energy for the reaction between LNCl and the SE of interest. We would like to highlight that a thermodynamic driving force for a chemical reaction (Δ*E*_D,mutual_ <0) between two SEs does not necessarily imply an immediate reaction upon contact; the diffusivity of elements in Li^+^-conducting SEs (excluding Li^+^) is limited at ambient temperatures so that an immediate solid-state reaction upon contact may be kinetically inhibited. Δ*E*_D,mutual_ can be treated as a propensity for reaction especially if the hybrid bilayer electrolyte is subjected to annealing/heating steps.^52^

Figure 5 shows that LNCl has the strongest propensity for chemical decomposition with LATP and sulfide-based SEs such as the argyrodite Li_6_PS_5_Cl and Li_3_PS_4_. With halide (Li_3_MCl_6_) electrolytes, the decomposition energy is reduced and seems to be mainly driven by the formation of strong M^3+^–N^3–^ bonds. The decomposition energy Δ*E*_D,mutual_ of Li_3_ScCl_6_ is 100 meV/atom larger than for Li_3_YCl_6_, which can be explained by a stronger Sc–N affinity compared to Y–N. The propensity for chemical decomposition is further reduced between LNCl and LIPON-type electrolytes and the garnet (LLZO) electrolyte. Figure 5 equally shows the pseudobinaries of Li_3_N and the SEs of interest (dashed lines). α-Li_3_N has a larger ion conductivity than LNCl (0.1–1 mS cm^–1^)^57,58^ and is equally thermodynamically stable against Li metal.^57^ Similar to LNCl, the applicability of α-Li_3_N is confined by its limited anodic limit (0.44 V).^57,59^ However, the driving force for chemical decomposition with common SEs is larger with Li_3_N by 50–200 eV/atom than with LNCl. This suggests a stronger propensity for decomposition at the Li_3_N|SE interface than at the LNCl|SE interface. Hybrid bilayer electrolytes are scarcely investigated but bear the potential to enable ASSBs.^60^ Beyond good ion conductivity (∼0.1 mS cm^–1^), the anolyte needs to balance chemical stability, contact stability, lithium dendrite suppression on the LM side, and compatibility with the catholyte. This complex interplay of chemical, mechanical, and microstructural properties of the anolyte motivates the exploration and optimization of different phases that are thermodynamically stable against LM such as the lithium nitride halides. However, the future practicality of LNCl will depend on whether its practical conductivity can be increased. Leveraging the findings of this study an investigation on compositional modifications of LNCl as well as the benefits of these materials for full batteries is underway.

## Conclusion

We investigated the fundamental Li diffusion mechanism in LNCl using AIMD simulations. We found that Li diffuses through the edge of edge-sharing tetrahedral Li sites. The propensity for jumps between two Li sites depends on the N/Cl composition of the tetrahedra enclosing the Li sites and of the bottleneck-edge composition. We found that jumps into Cl_4_ Li sites and jumps through Cl–Cl edges have the largest *E*_a,jump_ values, indicating that they are unfavorable compared to other jump events. Our EIS experiments confirmed the previously reported Li conductivity of 1 × 10^–3^ mS cm^–1^ and an *E*_a_ of 0.47 eV. Much faster Li motion was detected with ^7^Li NMR (>0.1 mS cm^–1^), with a lower *E*_a_ ∼ 0.25 eV. We propose that the fast Li motion may arise from readily occurring jumps with low *E*_a,jump_ values and that the long-range conductivity probed by EIS is limited by jumps with large *E*_a,jump_ values. Introducing a 4 h mechanochemical milling step after solid-state synthesis improves the RT conductivity of LNCl by an order of magnitude to 0.01 mS cm^–1^ and lowers the activation energy to 0.42 eV. It was shown that LNCl can be synthesized mechanochemically without any annealing step, and these samples reached conductivities of 0.015 mS cm^–1^. Reannealing of milled LNCl samples at 600 °C eliminates the beneficial effects of milling, as the conductivity is reduced and the activation energy increased to values obtained for nonmilled samples. Our experiments showed that the anodic limit of LNCl is ∼0.6 V (vs Li^+^/Li), which is significantly lower than the previously reported >2 V (vs Li^+^/Li) and matches our first principles thermodynamic calculations. We established that the anodic limit of LNCl confines its potential role in ASSBs to an artificial buffer layer between LM and other highly-conducting SEs, where the thermodynamic stability of LNCl against LM may be beneficial. Our calculations show that from a thermodynamic viewpoint, LNCl is chemically better compatible with common highly-conducting SEs than Li_3_N. Future investigations on LNCl should focus on different synthesis approaches of LNCl, how they affect subordering of the N/Cl anionic framework, and how this may potentially affect Li ion conductivity. Additionally, doping strategies have previously been investigated^37^ and should be further explored.

## Methodology

### Synthesis

*LNCl-I*: the synthesis precursors are LiCl (Sigma-Aldrich, 99%) and Li_3_N (Sigma-Aldrich, >99.5%). Stoichiometric amounts of the precursors with 10% wt excess Li_3_N were milled in a planetary ball mill (jar: ZrO_2_, 45 mL) with 10 mm ZrO_2_ balls and a ball/powder ratio of 13 at 270 rpm for 4.5 h (5 min milling; 15 min pause) to ensure good mixing of the precursors. Subsequently, the precursor mix was pressed into a pellet (1.3 tons) and transferred to an airtight Cu crucible. The crucible was placed in a furnace and heated at a rate of 300 °C/h to 600 °C, maintained at this temperature for 3 h, and then air quenched. *LNCl-I-BM*: To obtain LNCl-I-BM, LNCl-I was transferred to a planetary ball mill (jar: ZrO_2_, 45 mL) with 1 mm ZrO_2_ balls and a ball/powder ratio of 25 and milled for 4 h (5 min milling; 5 min pause). After each hour of milling, all powder was removed from the inner walls of the jar to ensure effective milling. *BM-LNCl*: stoichiometric amounts of LiCl and Li_3_N with 10% wt excess Li_3_N were milled in a planetary ball mill (jar: ZrO_2_, 45 mL) with 10 mm ZrO_2_ balls and a ball/powder ratio of 13 at 600 rpm for 10 h (5 min milling; 5 pauses). All preparation steps were done in an argon atmosphere (H_2_O < 1 ppm, O_2_ < 1 ppm). *LNCl-I in contact with molten Li*: LNCl-I was placed between two LM disks in a W crucible, which was sealed in a quartz ampoule. The ampoule was exposed to 210 °C for 2 h and then removed from the furnace.

### Electrochemical Characterization

*EIS*: pellets of the LNCl probes were pressed (3.2 tons) into custom-made solid-state lab cells. This yielded pellets of >85% densification; to determine this, the effective pellet density was calculated using the pellet thickness (measured with a digital caliper) and the diameter of the pellets. The effective pellet density was then divided by the crystallographic density to obtain the percentage of densification. The cell configuration was SS|LNCl|SS (SS = stainless steel). AC impedance was performed with an Autolab (AUT86298) in the frequency range of 10 MHz to 0.1 Hz with a voltage amplitude of 10 mV. *Galvanostatic measurements*: Galvanostatic measurements were also performed with an Autolab (AUT86298). To measure the anodic limit of LNCl, Li|LNCl-I-BM|LNCl-I-C was used. To make the LNCl-I-C composite cathode, a mixture of LNCl-I, Super P, and carbon-nanotubes with a weight ratio of 0.7:0.15:0.15 was milled in a planetary ball mill (jar: ZrO_2_, 45 mL) with 10 mm ZrO_2_ balls and a ball/powder ratio of 30 at 400 rpm for 2 h (5 min milling; 5 min pause). After the first hour of milling, all powder was removed from the inner walls of the jar to ensure good mixing. Li|LNCl-I-BM|LNCl-I-C cells were assembled by pressing an LNCl-I-BM pellet (130 mg, 3.2 tons), and subsequently the LNCl-I-C composite (15 mg, 3.2 tons) on top of it. Finally, a Li disk was placed on the opposite side of the LNCl-I-BM pellet. LNCl-I-BM was used as the SE because it is 10 times more conductive than LNCl-I, so voltage drops in the SE become negligible (<0.01 V) at the current used 0.5 μA (0.64 μA cm^–2^). *Conductivity measurements at different temperatures for Arrhenius fits*: SS|LNCl|SS cells were kept at 30 °C for 1 h, then heated for 5 min to 50 °C and kept at this temperature for 30 min, followed by heating to 60 °C in 5 min and maintaining the temperature for 30 min. This procedure was continued up to 100 °C. The EIS obtained at the end of the 30 min temperature plateaus was used for Arrhenius fits.

### Solid State NMR

Solid state NMR measurements were done on a Bruker Ascend 500 MHz spectrometer with a ^7^Li resonance frequency of 194.381 MHz. The 90° pulse length was typically 2.7–2.8 μs. Air-sensitive LNCl probes were sealed in 4 mm diameter Teflon rotors in an Ar glove box. The variable temperature ^7^Li NMR experiments was conducted statically without MAS in a temperature range from −120 to 160 °C. The chemical shifts were referenced to a 0.1 M aqueous LiCl solution.

### X-ray Diffraction

Powder diffractograms were collected in the 2θ range 10–100° using Cu Kα X-rays (1.54 Å, 45 kV, 40 mA) on a PANalytical X’Pert Pro X-ray diffractometer. The air-sensitive LNCl probes were loaded into air-tight holders in an Ar glovebox prior to the measurements. A LaB_6_ NIST (NIST660c) standard was employed to calibrate instrumental broadening. The JANA2006 program^61^ was used for LeBail and Rietveld refinements and the Williamson-Hall analysis.

### Computational Details

All DFT calculations were performed with the Vienna ab-initio simulation package VASP with computational settings consistent with those used in the Materials Project database.^53^ Calculations were done on a 2 × 2 × 2 LNCl supercell. Because of the partial occupancies in LNCl, different atomic arrangements were generated. The starting point was an initial guess of a 2 × 2 × 2 supercell, with all Li positions occupied (real supercell stoichiometry, Li_64_N_11_Cl_21_). The N/Cl arrangement was optimized by minimizing the electrostatic energy using the *OrderDisorderStandardTransformation* tool as implemented in pymatgen.^62^ Subsequently, for the 20 arrangements with the lowest electrostatic energy, 20 further supercells were generated by removing Li, again minimizing the electrostatic energy with the *PartialRemoveSpeciesTranfomation* tool as implemented in pymatgen.^62^ From this pool of 400 supercells, the 40 supercells with the lowest electrostatic energy were relaxed with DFT. Additionally, >10,000 supercells were generated randomly. The 40 “random” supercells with the lowest electrostatic energy were relaxed with DFT. The supercell with the lowest internal energy, as calculated by DFT, was taken. and the N/Cl arrangement was reoptimized, minimizing the electrostatic energy, and the 40 supercells with the lowest electrostatic energy were relaxed with DFT. Of all the supercells generated, the one with the lowest internal energy, as calculated by DFT, was adopted as the model supercell. The stoichiometry of the supercells was always Li_4.82_NCl_1.91_ (Li_53_N_11_Cl_21_) and approximates the Li_5_NCl_2_ (Li_53.333_N_10.666_Cl_21.333_) stoichiometry as well as possible given the supercell-size constraints in AIMD simulations. The electrochemical stability window and the pseudobinaries were calculated, as described in the work by Zhu, He, and Mo.^52^ For the pseudobinaries, the SE phase compositions with energies above the hull set to zero were added to the materials project phase space. Subsequently, *E*_D,mutual_, as defined by Zhu, He, and Mo^52^ was calculated between LNCl and the respective SEs. AIMD simulations were done in the NVT ensemble with 2 fs time steps. The k-point grid used was 1 × 1 × 1, and the energy cutoff was 400 eV. For the AIMD simulations, the Li pseudopotential was changed from Li_sv (which was used for relaxations) to Li, as this enables the use of a lower energy cutoff. The simulation time was >200 ps for every AIMD simulation, and the error on the obtained diffusivities was estimated, as shown by He and Mo et al.^35^ The dissection of AIMD simulations into individual jump events and subsequent analysis of the prefactor frequency, jump-diffusivity, jump frequencies, and individual *E*_a,Jump_ values was done as first described by de Klerk and Wagemaker;^30^ a comprehensive account can be found in ref (30), but crucial aspects for the understanding of the reported data is presented here. *Calculation of E*_*a,jump*_*values between two sites*: The sites are defined around the 0 K equilibrium positions of the Li ions. At every simulation step, it is recorded at which site each Li ion is located or whether it is currently between two sites. From this information, the jump frequency between two sites v_A→B_ can be calculated according to eq 8
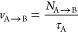
8where *v*_A→B_ is the jump frequency for jumps from site A to site B, *N*_A→B_ is the number of recorded jumps from A to B, and τ_A_ is the time of occupation of site A. *E*_a,jump_ is then obtained from eq 1. *Calculation of jump diffusivities and tracer diffusivities D*_*J*_*and D*_*tr.*_: *D*_J_ and *D*_tr._ are in principle calculated with the same formula
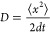
9where ⟨*x*^2^⟩ is the mean square displacement of the lithium ions, d is the dimensionality of diffusion, and *t* is the simulation time. *D*_J_ and *D*_tr_ differ in the way ⟨*x*^2^⟩ is calculated. For *D*_tr_, ⟨*x*^2^⟩ is directly obtained from the AIMD simulation. For *D*_J_, ⟨*x*^2^⟩ is calculated by summing up the jump distances and averaging over the number of Li ions
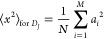
10where *N* is the total number of diffusing ions, *a*_A→B_ is the jump distance of a generalized jump-event “*i*”, and *M* is the total number of jumps observed. Combining eqs 9 and 10 yields eq 4. *Calculation of the relative site energies*: LNCl intrinsically contains Li vacancies. The site-energy difference between site A and site B was obtained by displacing a Li in site A to an empty site B. The energy difference between the two supercells was interpreted as the site-energy difference between sites A and B. The average site-energy difference between sites A and B was calculated from 10 such displacements.

## Supporting Information Available

Comparison of computational and experimental lattice parameters; LNCl supercell and Li–N and Li–Cl stationary RDF; Arrhenius relationship obtained from AIMD for LNCl; table with tracer and jump diffusivities obtained from AIMD at different temperatures; table summarizing the pseudobinaries between LNCl and other electrolytes; statistical details on the supercell site and bottleneck distribution; XRD and refinement of LNCl-I and LNCl-I-BM; EIS of LNCl-I at −30 C; Williamson Hall analysis; Li|LNCl-I-BM|Li symmetric cell; and Arrhenius relationships of LNCl after different annealing protocols (PDF)
