# Supplementary material for: Li5NCl2: A Fully-Reduced, Highly-Disordered Nitride-Halide Electrolyte for Solid-State Batteries with Lithium-Metal Anodes
Source: ACS Appl Energy Mater. 2023 Jan 27;6(3):1661–72. doi: 10.1021/acsaem.2c03551 (PMC9930088; doi:10.1021/acsaem.2c03551)
Supplement: Supplementary file 1 — ae2c03551_si_001.pdf [file ae2c03551_si_001.pdf]

Supporting information

Li_5NCl_2: A fully-reduced, highly-disordered nitride-halide electrolyte for solid-state batteries with lithium-metal anodes

Victor Landgraf[a], Theodosios Famprikis[a], Swapna Ganapathy[a], Lars Johannes Bannenberg[a], Joris de Leeuw[a] Marnix Wagemaker[a*]

[a] Faculty of Applied Sciences, Delft University of Technology, 2628 Delft, The Netherlands

*m.wagemaker@tudelft.nl

Table S1. Comparison between experimental lattice parameters and the model supercell employed in this investigation. Note of caution, the experimental lattice parameters reported in previous work and the one obtained in our XRD measurements are multiplied by two here, to be comparable with the lattice parameter of the 2x2x2 LNCl supercells employed in computational work. We would also like to mention that in references 1 ,2 and 3 the authors assumed a $Li_9N_2Cl_3$ stoichiometry which affects their reported densities.

	a [Å]	b [Å]	c [Å]	Volume [Å^3]	Density [g cm^{-3}]
**Previous experimental reports** [1 2 4]	10.772<br>10.814<br>10.832	10.772<br>10.814<br>10.832	10.772<br>10.814<br>10.832	1249.94<br>1264.617<br>1270	1.67<br>1.65<br>1.67
**Previous computational report (DFT)** [3]	10.205	11.406	10.296	1198	1.74
**From our XRD experiments**	10.792	10.792	10.792	1256.915	1.69
**Model supercell employed in this work (DFT)**	10.858	10.556	10.913	1250.727	1.68*
**Difference between our experiments and our model supercell [%]**	-0.61	+2.23	-1.10	+0.49	+0.59

*The actual supercell stoichiometry, $Li_{4.82}NCl_{1.91}$ ($Li_{53}N_{11}C_{21}$), was used to calculate this density.

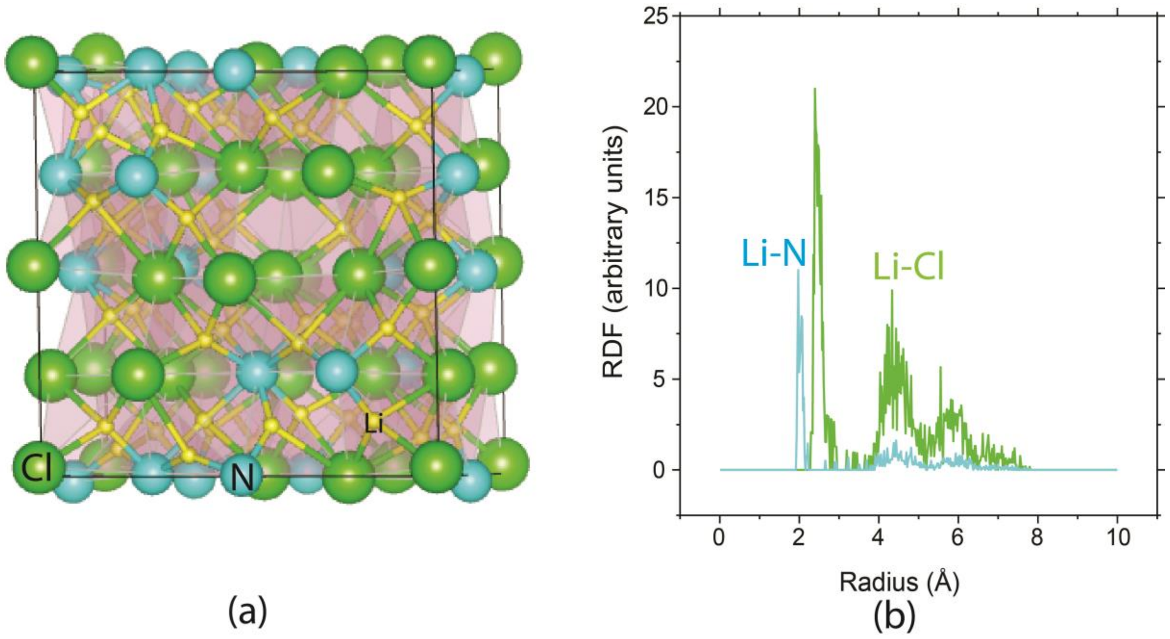

Figure S1. a) LNCl model supercell. Li displacement from the ideal tetrahedral position can be well observed. b) RDF of Li-Cl and Li-N for the stationary LNCl supercell shown in a. The peak at lower radii in the Li-N RDF indicates the displacement of Li site position towards N within the tetrahedra.

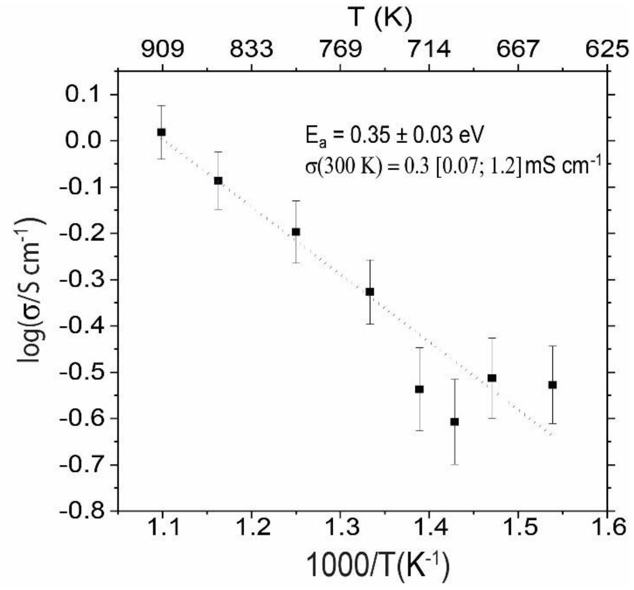

Figure S2. Arrhenius plot obtained from the tracer diffusivities D_{tr} calculated with AIMD simulations. The error on the diffusivities were calculated as done by He and Mo.[5]

Table S2. This table lists the tracer and jump diffusivities obtained from AIMD simulations at different temperatures. The correlation factor at different temperatures is also listed.

Temperature	650 K	680 K	700 K	720 K	800 K	860 K	910 K	Average
10^{-10} * D_{tr} [m^2 s^{-1}]	2.40	2.64	2.19	2.65	6.40	8.94	12.0	-
10^{-10} * D_J [m^2 s^{-1}]	6.26	7.54	7.45	8.58	13.60	17.20	24.0	-
$f = D_{tr}/ D_J$	0.38	0.35	0.29	0.31	0.47	0.52	0.50	0.4 ± 0.1

Table S3. This table contains the data obtained from the pseudo-binaries between LNCl and common SE.

SE	Molar fraction of Li_5NCl_2 in Li_5NCl_2 - SE	Phase equilibria	$\Delta E_{D, mutual}$ (eV/atom)
**Li_6PS_5Cl**	1	Li_5NCl_2	0
	0.711111111	Li_7PN_4,LiCl, Li_2S	-0.251
	0.551724138	$LiPN_2$, LiCl, Li_2S	-0.302
	0	Li_6PS_5Cl	0
**LIPON $Li_{2.88}N_{0.14}O_{3.73}$**	1	Li_5NCl_2	0
	0.799	Li_7PN_4, Li_2O, LiCl	-0.137
	0.657	Li_2O, $LiPN_2$, LiCl	-0.200
	0.470	Li_2PNO_2, Li_2O, LiCl	-0.088
	0	$Li_{2.88}N_{0.14}O_{3.73}$	0
**Li_3PS_4**	1	Li_5NCl_2	0
	0.2	Li_7PN_4, LiCl, Li_2S	-0.267
	0.3	$LiPN_2$, LiCl, Li_2S	-0.338
	1	Li_3PS_4	0
**Li_3ScCl_6**	1	Li_5NCl_2	0
	0.385	Li_3ScN_2, LiCl	-0.195
	0.444	ScN ,LiCl	-0.268
	0	Li_3ScCl_6	0
**Li_3YCl_6**	1	Li_5NCl_2	0
	0.385	Li_3YN_2, LiCl	-0.145
	0.556	YN, LiCl	-0.206
	0.714	Y_2NCl_3, LiCl	-0.138
	0	Li_3YCl_6	0
**LLZO ($Li_7La_3Zr_2O_{12}$)**	1	Li_5NCl_2	0
	0.7	Li_2O, Li_2ZrN_2, LiCl, LaN	-0.093
	0.571	Li_2O, La_2O_3, Li_2ZrN_2, LiCl	-0.094
	0	$Li_7La_3Zr_2O_{12}$	0

Table S4. This table contains the data obtained from the pseudo-binaries between Li_3N and common SE.

SE	Molar fraction of Li_3N in Li_3N-SE	Phase equilibria	$\Delta E_{D,\ mutual}$ (eV/atom)
**Li_6PS_5Cl**	1	Li_3N	0
	0.606	Li_7PN_4, Li_4NCl, Li_2S	-0.350
	0.552	Li_7PN_4, LiCl, Li_2S	-0.396
	0.380	$LiPN_2$, LiCl, Li_2S	-0.421
	0	Li_6PS_5Cl	0
**LIPON $Li_{2.88}N_{0.14}O_{3.73}$**	1	Li_3N	0
	0.666	Li_7PN_4, Li_2O	-0.235
	0.490	Li_2O, $LiPN_2$	-0.184
	0.307	Li_2PNO_2, Li_2O	-0.119
	0	$Li_{2.88}N_{0.14}O_{3.73}$	0
**Li_3PS_4**	1	Li_3N	0
	0.667	Li_7PN_4, Li_2S	-0.267
	0.5	$LiPN_2$, Li_2S	-0.338
	1	Li_3PS_4	0
**Li_3ScCl_6**	1	Li_3N	0
	0.761	Li_3ScN_2, Li_4NCl	-0.129
	0.444	Li_3ScN_2 ,LiCl	-0.286
	0.286	ScN ,LiCl	-0.348
	0	Li_3ScCl_6	0
**Li_3YCl_6**	1	Li_3N	0
	0.238	Li_3YN_2, Li_4NCl	-0.098
	0.556	Li_3YN_2, LiCl	-0.216
	0.714	YN, LiCl	-0.268
	0.833	Y_2NCl_3, LiCl	-0.162
	0	Li_3YCl_6	0
**LLZO ($Li_7La_3Zr_2O_{12}$)**	1	Li_3N	0
	0.538	Li_2O, Li_2ZrN_2, LaN	-0.149
	0.400	Li_2O, La_2O_3, Li_2ZrN_2	-0.136
	0	$Li_7La_3Zr_2O_{12}$	0

Statistical details on the model LNCl supercell employed in this study

A jump-E_a value for a jump from A to B is obtained by counting the jumps from A to B. The number of jumps $N_{A\text{->}B}$ is then divided by the occupation time of site A τ_A. Using the equations 1 and 2 below, the jump-E_a values are obtained:

$$v_{A\to B} = \frac{N_{A\to B}}{\tau_A} \qquad (1)$$

$$E_{a,\ A\to B} = -\mathrm{k_b T ln}\left(\frac{\mathrm{v_{A\to B}}}{\mathrm{v^*}}\right) \qquad (2)$$

More than one A-B pair is required to determine the uncertainty on the average $E_{a,\ A\text{->}B}$ value. Table S5 below shows the occurrence of A-B pairs in the model supercell.

Table S5. From this table one obtains how many neighbouring A-B sites are 'connected' by Cl-Cl, Cl-N, and N-N bottlenecks respectively, and how many A-B pairs can be found in the model supercell.

Site pairs	Bottleneck that connects the pair			Sum of pairs
	Cl-Cl	Cl-N	N-N	
Cl_4 - Cl_4	5	/	/	5
Cl_4 - Cl_3N_1	31	/	/	31
Cl_4 - Cl_2N_2	7	/	/	7
Cl_4 - Cl_1N_3	/	/	/	/
Cl_3N_1 -Cl_3N_1	25	17	/	42
Cl_3N_1 -Cl_2N_2	9	43	/	52
Cl_3N_1 -Cl_1N_3	/	13	/	13
Cl_2N_2 -Cl_2N_2	2	16	6	24
Cl_2N_2 -Cl_1N_3	/	5	8	13
Cl_1N_3 - Cl_1N_3	/	0	5	5
SUM	79	94	19	192

Note: "/" means that such sites cannot be neighbouring. For example, because the tetrahedra in LNCl are edge-sharing, two neighbouring Cl_4 sites cannot be "connected" by a Cl-N or N-N bottleneck. "0" means that the sites could in principle be connected by the bottleneck in question but that such a case is not represented in the model supercell. $E_{a,\ jump}$ values are obtained for every individual pair of sites and the ones reported are averaged over all pairs of sites.

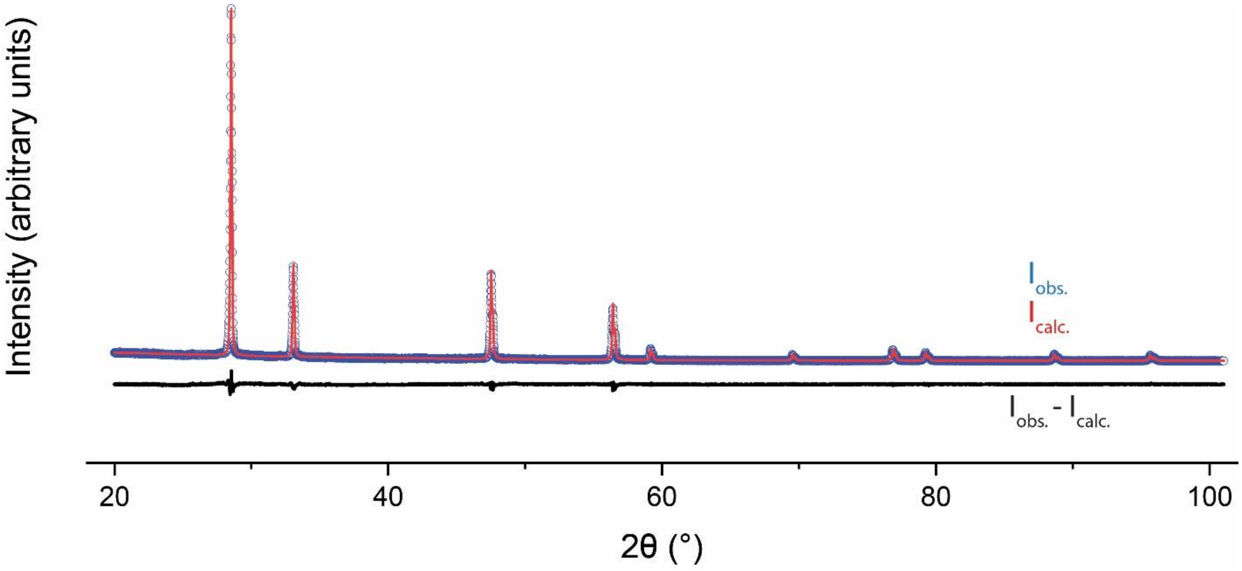

Figure S3. XRD of LNCl-I. This is also shown in Figure 3 of the main text. a = 5.39(6) Å. X-ray reliability parameters: R_p = 5.69,R_{wp} = 7.39, χ^2 = 2.3

Table S6. Structural parameters from the Rietveld refinement of LNCl-I.

Atom	x	y	z	Wyckoff	Occupancy	U_{iso}
Li	0.25	0.25	0.25	8c	5/6	0.14(0)
N	0	0	0	4a	1/3	0.18(0)
Cl	0	0	0	4a	2/3	0.02(5)

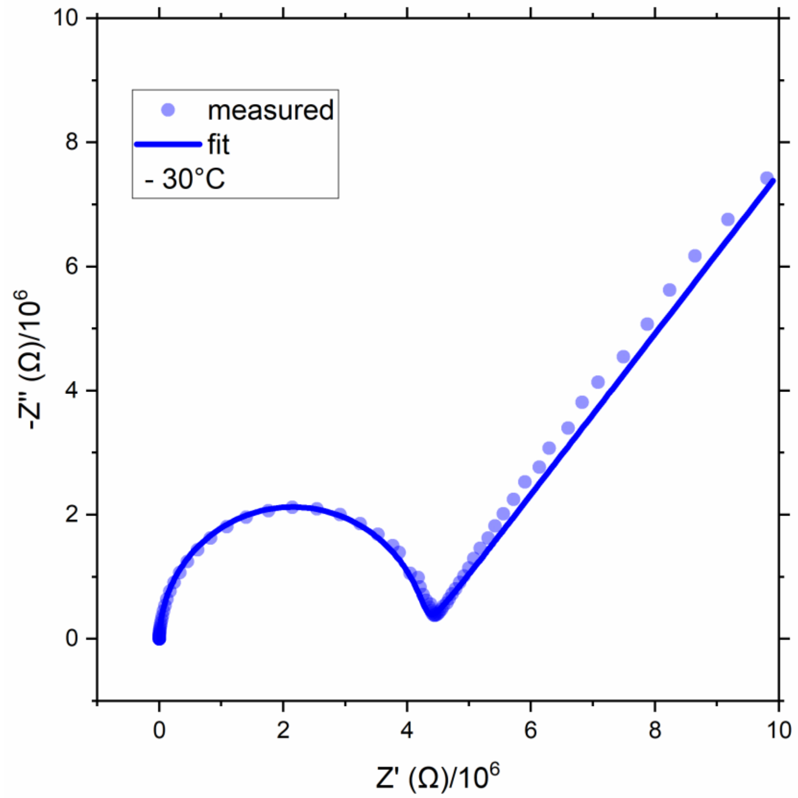

Figure S4. EIS of LNCl-I at -30 °C fitted to a p(R-CPE)-CPE circuit.

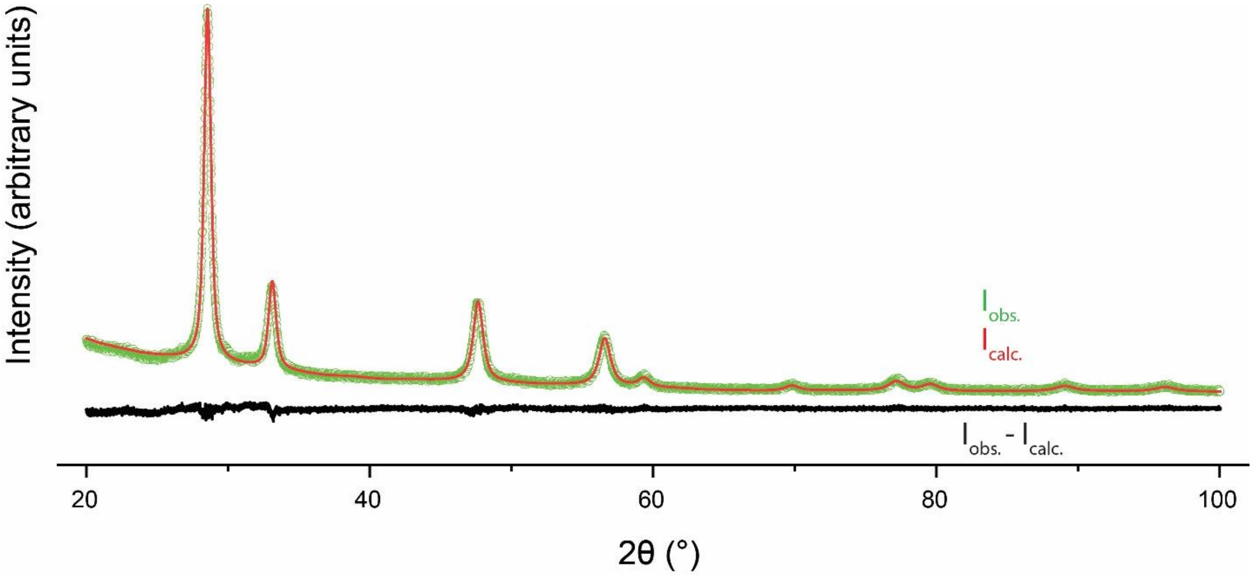

Figure S5. XRD of LNCl-I-BM. This is also shown in Figure 3 of the main text. a = 5.37(6) Å. X-ray reliability parameters: R_p = 5.08,R_{wp} = 6.46, χ^2 = 3.57

Table S7. Structural parameters from the Rietveld refinement of LNCl-I-BM.

Atom	x	y	z	Wyckoff	Occupancy	U_{iso}
Li	0.25	0.25	0.25	8c	5/6	0.12(1)
N	0	0	0	4a	1/3	0.14(0)
Cl	0	0	0	4a	2/3	0.02(4)

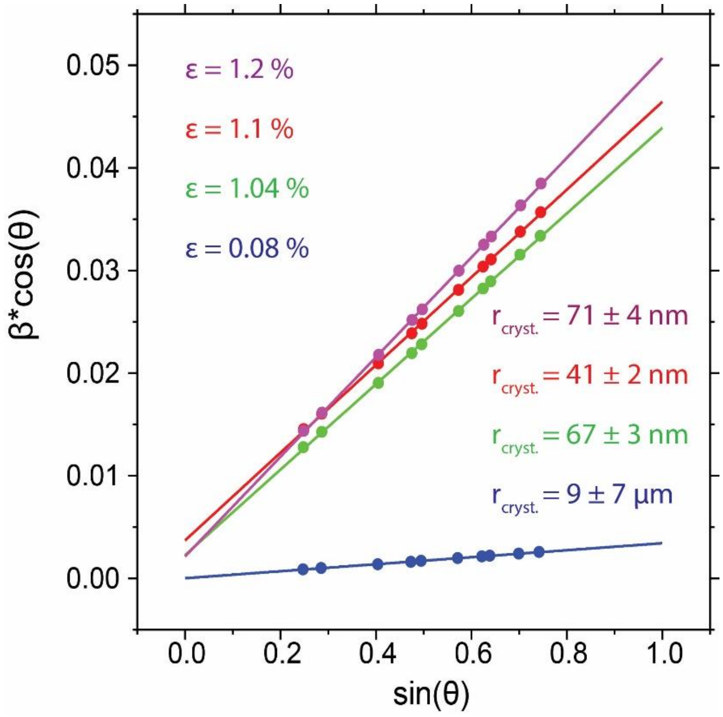

Figure S6. Williamson-Hall analysis of the diffractograms of (blue) LNCl-I, (green) LNCl-I-BM, (red) LNCl-I-BM-8h, (violet) LNCl-I-BM-12h. β here is the peak-width in the diffractograms. These were obtained with the Jana2006 software. [6]

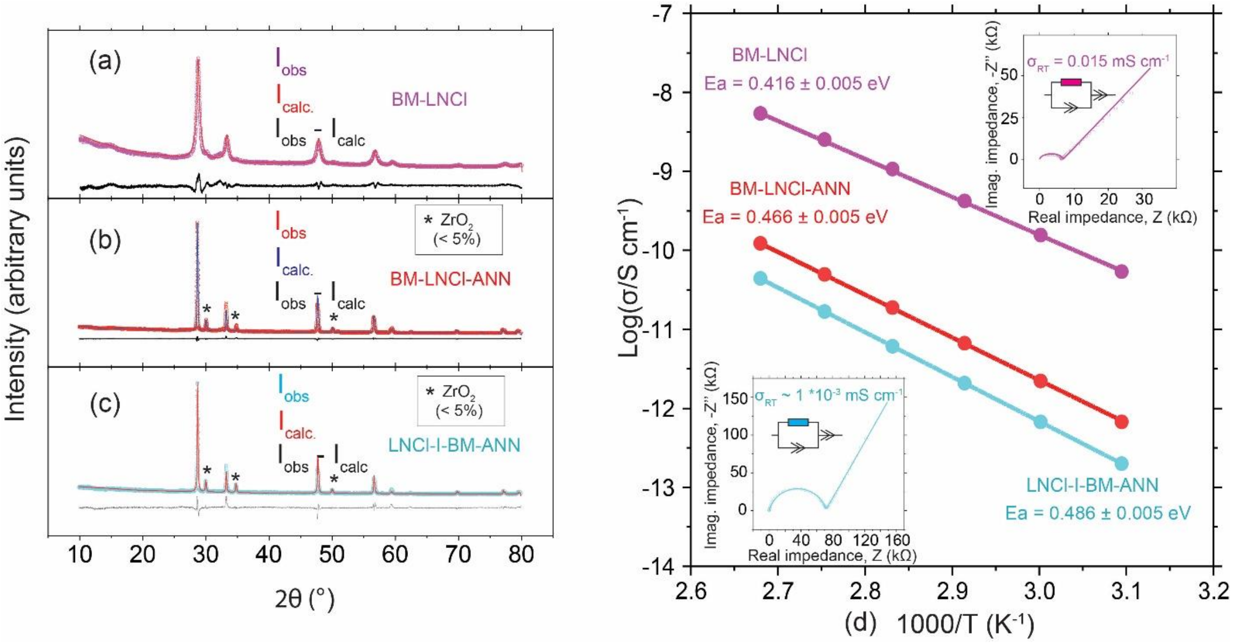

Figure S7. (a) , (b), (c) are X-ray diffractograms of BM-LNCl, BM-LNCl-ANN, LNCl-I-BM-ANN. A small ZrO_2 impurity likely coming from the milling medium is marked with * (d) Arrhenius plots for BM-LNCl, BM-LNCl-ANN, LNCl-I-BM-ANN. The inset at the lower left shows the EIS of LNCl-I-BM-ANN and the one at the top right shows the EIS of BM-LNCl. (ANN is an abbreviation for annealed. E.g. BM-LNCl-ANN refers to the BM-LNCl sample that is annealed at 600 °C for 3 h and then air quenched as described in the main text).

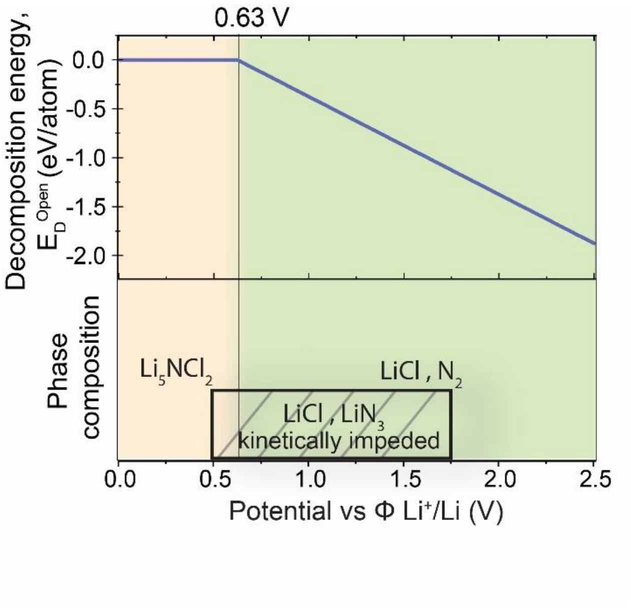

Figure S8. Phase equilibria of the Li_xNCl_2 {x∈R|x≥0} phase space at different potentials ϕ vs Li^+/Li. Additionally this figure shows the decomposition energy $E_{D,\,open}$ as defined by Zhu and Mo [7] of LNCl at different ϕ. The difference here to figure 4a is that LiN_3 was removed from the phase space obtained from the materials project.[8] As a consequence the stability window is shifted up slightly from 0.50 V to 0.63 V. Investigating this was motivated by the following reasoning. Decomposition of LNCl to LiN_3 necessitates a complex rearrangement of the anionic framework to from the $[N_3]^-$ moieties present in LiN_3. Given the complexity of this rearrangement and the likely limited RT diffusivity of N^{3-} and Cl^- in LNCl, it is conceivable that the formation of LiN_3 may be kinetically impeded even at very low current densities and that LNCl "directly" decomposes to LiCl and N_2.

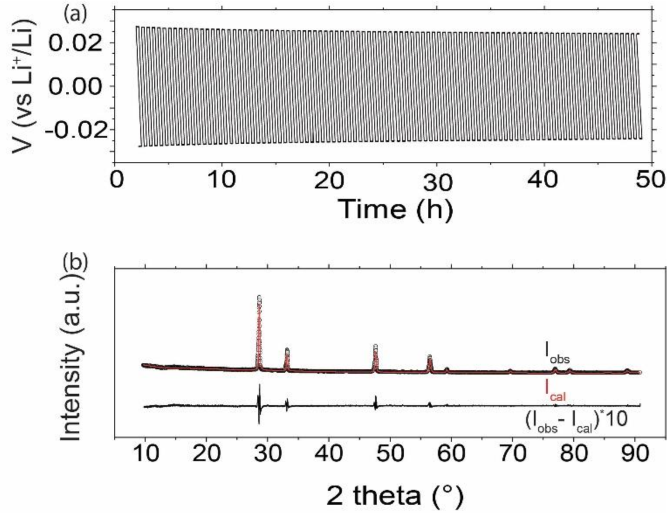

Figure S9. (a) Example of the cycling of a Li|LNCl-I-BM|Li cell. Applied current density: 0.01 mA cm^{-2}. Time for one cycle: 30 min. This cell was cycled at 50 °C to better control temperature. No increase in the cell voltage is observed over time which indicates excellent compatibility of LNCl and Li-metal (LM). A small decrease (from 26 to 24 mV) in the cell voltage is observed at the early stages of cycling which may be a consequence of improved contact between LM and LNCl establishing during the first cylces. (b) X-ray diffractogram of LNCl-I after in contact with molten LM at 210 °C for 2h. No other phases besides LNCl are observed that would indicate decomposition of LNCl in contact with LM. The difference plot was multiplied by 10 to make better visible the differences between I_{obs} and I_{calc}.

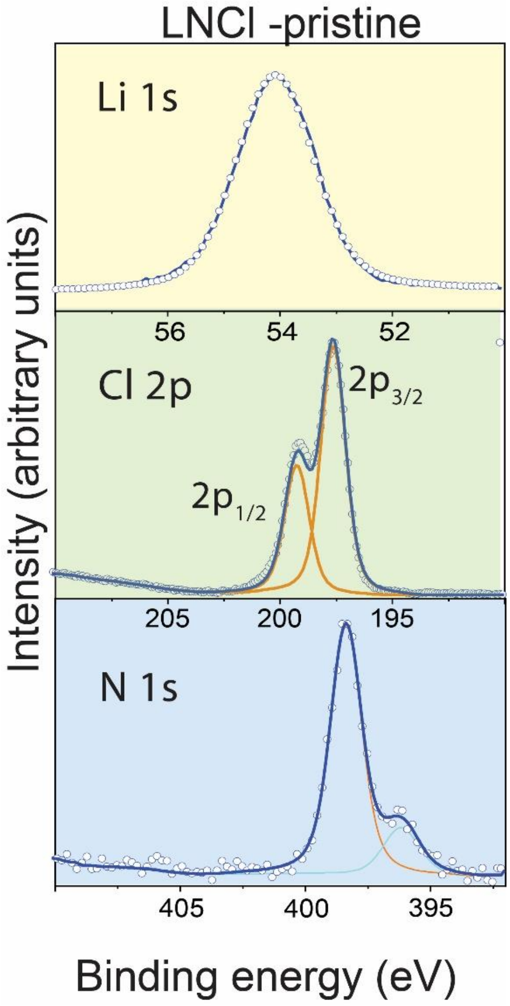

Figure S10. Li1s Cl2p and N1s XPS spectra of LNCl. A second peak is observed in the N1s spectrum (small blue peak). Comparison with the XPS NIST database[9] the binding energies of this peak match different chemical compositions that can be summarized by the general formula $(C_xH_yN_z)_n$. The presence of this second small contribution in the XPS spectrum suggests an impurity on the surface of the LNCl particles. This impurity may have been introduced during the transfer from the glovebox to the XPS machine inside the vacuum transfer unit.

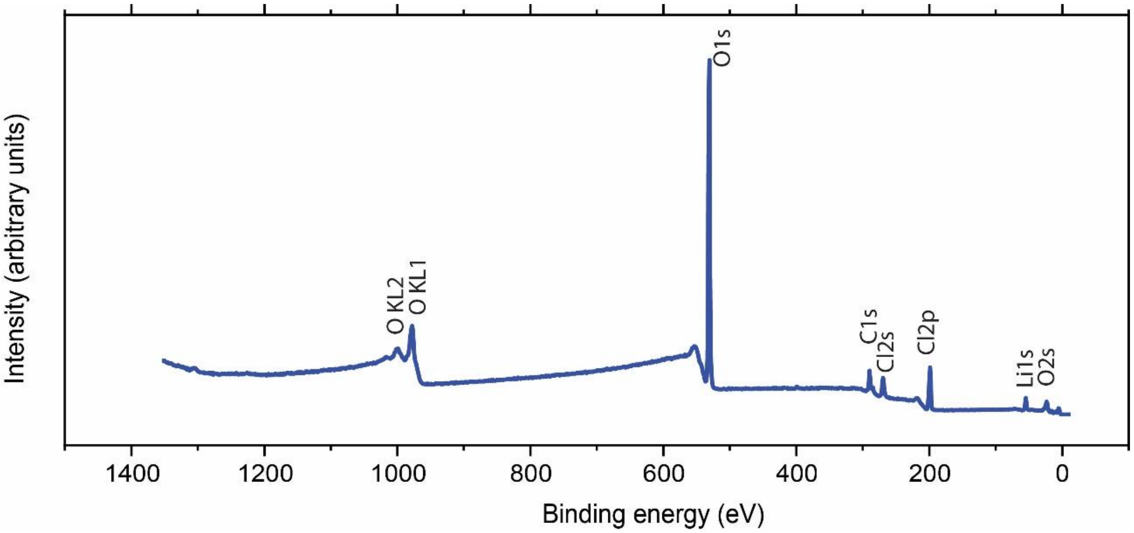

Figure S11. XPS survey scan of LNCl.

Experimental details of X-ray photon spectroscopy. XPS measurements were performed with a ThermoFisher K-Alpha spectrometer to investigate the chemical state of the elements present. The spectrometer is equipped with a focused monochromatic Al kα source (1486.6 eV) anode operating at 36 W (12 kV, 3mA), a flood gun operating at 1V, 100µA, and the base pressure in the analysis chamber is approximately $2{\cdot}10^{-9}$ mbar. The spot-size is approximately 800 x 400 µm^2. The pass energy of the analyzer was set to 50 eV. The samples were transferred from the glove box to the spectrometer inside a dedicated Thermofisher vacuum transfer module to avoid air-exposure. In the analysis, the binding energy was corrected for the 0.5 eV charge shift by taking the primary C1s hydrocarbon peak at *BE* = 284.8 eV as a reference. The peaks were fitted using 70% Gaussian and 30% Lorentzian line shapes (weighted least-squares fitting method) and nonlinear Shirley-type background using the ThermoFisher Avantage software.

(1) Hartwig, P.; Rabenau, A.; Weppner, W. Phase Equilibria and Theromdynamic Properties of the Li-N-Cl, Li-N-Br and Li-N-I Systems. **1981**, *80*, 81–90.

(2) Sattlegger, H.; Hahn, H. Uber Das System Li , N / LiCl. **1964**, *173*, 1–7.

(3) Galvez-Aranda, D. E.; Seminario, J. M. Ab Initio Study of the Interface of the Solid-State Electrolyte Li 9 N 2 Cl 3 with a Li-Metal Electrode . *J. Electrochem. Soc.* **2019**, *166* (10), A2048–A2057.

(4) Marx, R.; Mayer, H. M. Preparation and Crystal Structure of Ordered and Disordered Lithium Nitride Dichloride, Li5NCl2. *J. Solid State Chem.* **1997**, *130* (1), 90–96.

(5) He, X.; Zhu, Y.; Epstein, A.; Mo, Y. Statistical Variances of Diffusional Properties from Ab Initio Molecular Dynamics Simulations. *npj Comput. Mater.* **2018**, *4* (1), 1–9.

(6) Petříček, V.; Dušek, M.; Palatinus, L. Crystallographic Computing System JANA2006: General Features. *Zeitschrift für Krist. - Cryst. Mater.* **2014**, *229* (5), 345–352.

(7) Zhu, Y.; He, X.; Mo, Y. First Principles Study on Electrochemical and Chemical Stability of Solid Electrolyte-Electrode Interfaces in All-Solid-State Li-Ion Batteries. *J. Mater. Chem. A* **2016**, *4* (9), 3253–3266.

(8) Jain, A.; Ong, S. P.; Hautier, G.; Chen, W.; Richards, W. D.; Dacek, S.; Cholia, S.; Gunter, D.; Skinner, D.; Ceder, G.; Persson, K. A. Commentary: The Materials Project: A Materials Genome Approach to Accelerating Materials Innovation. *APL Mater.* **2013**, *1* (1), 001002.

(9) *NIST X-ray Photoelectron Spectroscopy Database, NIST Standard Reference Database Number 20*.
